# Genome-wide upstream motif analysis of *Cryptosporidium parvum* genes clustered by expression profile

**DOI:** 10.1186/1471-2164-14-516

**Published:** 2013-07-29

**Authors:** Jenna Oberstaller, Sandeep J Joseph, Jessica C Kissinger

**Affiliations:** 1 Center for Tropical and Emerging Global Diseases, University of Georgia, Athens, GA 30602, USA; 2 Department of Genetics, University of Georgia, Athens, GA 30602, USA; 3 Institute of Bioinformatics, University of Georgia, Athens, GA 30602, USA

**Keywords:** Apicomplexa, Transcription, Gene regulation, Motif, AP2, E2F, G-box

## Abstract

**Background:**

There are very few molecular genetic tools available to study the apicomplexan parasite *Cryptosporidium parvum*. The organism is not amenable to continuous *in vitro* cultivation or transfection, and purification of intracellular developmental stages in sufficient numbers for most downstream molecular applications is difficult and expensive since animal hosts are required. As such, very little is known about gene regulation in *C. parvum*.

**Results:**

We have clustered whole-genome gene expression profiles generated from a previous study of seven post-infection time points of 3,281 genes to identify genes that show similar expression patterns throughout the first 72 hours of *in vitro* epithelial cell culture. We used the algorithms MEME, AlignACE and FIRE to identify conserved, overrepresented DNA motifs in the upstream promoter region of genes with similar expression profiles. The most overrepresented motifs were E2F (5′-TGGCGCCA-3′); G-box (5′-G.GGGG-3′); a well-documented ApiAP2 binding motif (5′-TGCAT-3′), and an unknown motif (5′-[A/C] AACTA-3′). We generated a recombinant *C. parvum* DNA-binding protein domain from a putative ApiAP2 transcription factor [CryptoDB: cgd8_810] and determined its binding specificity using protein-binding microarrays. We demonstrate that cgd8_810 can putatively bind the overrepresented G-box motif, implicating this ApiAP2 in the regulation of many gene clusters.

**Conclusion:**

Several DNA motifs were identified in the upstream sequences of gene clusters that might serve as potential *cis*-regulatory elements. These motifs, in concert with protein DNA binding site data, establish for the first time the beginnings of a global *C. parvum* gene regulatory map that will contribute to our understanding of the development of this zoonotic parasite.

## Background

The AIDS-related protist parasite *Cryptosporidium parvum* primarily infects the microvillous border of the intestinal epithelium, and to a lesser extent extraintestinal epithelia, causing acute gastrointestinal disease in a wide range of mammalian hosts. The first case of human *Cryptosporidium* infection was reported in 1976 [1], and only seven additional cases were documented before 1982 [2]. Since then the number of cases identified has increased dramatically, largely due to the recognition of a life-threatening form of infection in immunocompromised individuals [3]. *Cryptosporidium* was also recently implicated as a significant pathogen contributing to moderate-to-severe diarrhea in children under two years of age in sub-Saharan Africa, second only to rotavirus [4]. Seroprevalence rates of 25-35% in the United States indicate that infection with *Cryptosporidium* is very common among healthy persons [5].

*C. parvum* has a complex, obligate-intracellular life cycle involving both asexual and sexual developmental stages. Transmission of *Cryptosporidium* occurs through the fecal-oral route where an infection is initiated by the ingestion of oocysts, which release sporozoites capable of invading intestinal epithelial cells. The parasite’s obligate intracellular developmental stages are exceedingly difficult to study. The volume of parasite material relative to host cell is vanishingly small. Each parasite is 5 μm or smaller (depending on lifecycle stage; [6]) in a host cell that is a hundred to thousand times larger in volume. Given these complications of size, the post-infection parasite cannot be isolated from host cells in sufficient numbers, nor can sufficient post-infection parasite protein or RNA be obtained for most downstream molecular applications. Currently, *C. parvum* is not amenable to continuous *in vitro* cultivation or genetic dissection [7,8].

Given the above-mentioned difficulties, transcriptional regulation in this parasite is largely unknown. Indeed, transcriptional regulation across the entire apicomplexan phylum is still poorly understood, though the combination of computational and bench analyses have yielded significant discoveries in the distantly related parasites *Plasmodium falciparum* and *Toxoplasma gondii*. Genome-wide scans of the phylum for proteins containing possible DNA-binding domains revealed several families of DNA-binding proteins including a significant expansion of the Apicomplexan AP2 (ApiAP2) family of transcriptional regulators [9]. Subsequent experimental analyses confirmed regulatory roles for several of these ApiAP2 proteins [10-12]. Campbell *et al.* (2010) [13] determined DNA-binding specificities for 20/27 identified members of this family in *P. falciparum* by generating recombinant ApiAP2 proteins and testing them on protein-binding microarrays (PBMs) [14]. These experiments identified binding site sequences matching several previously determined *Plasmodium cis*-elements. Militello *et al.* (2004) computationally predicted a *cis*-regulatory element in the upstream sequences of 8/18 *P. falciparum* heat shock genes (called the G-Box) and subsequently demonstrated the importance of this element through transient transfections and mutational analyses [15]. Similarly, Young *et al.* (2008) predicted several *cis* regulatory elements upstream of *Plasmodium* genes clustered based on similarity of gene expression profile (21 clusters total) and demonstrated the regulatory importance of one of the predicted elements (PfM18.1, 5′-GTGCA-3′) *in vitro*[16]. Elemento *et al*. (2007) developed a powerful bioinformatic approach taking advantage of mutual information (expression information and overrepresentation of short DNA sequences upstream of potentially co-regulated genes) to predict several additional putative *cis*-regulatory elements [17]. The fact that Campbell *et al.* (2010) could identify specific *trans* factors that bound many of these motifs [13] confirms the power of computational methods to predict *cis*-regulatory elements in *Plasmodium*.

Computational methods have been used successfully to predict regulatory elements across the apicomplexan phylum, though unlike in *Plasmodium* we rarely know which, if any, *trans* factors bind these elements. In *Toxoplasma gondii*, Mullapudi *et al*. (2009) identified putative *cis*-regulatory elements present upstream of functionally related groups of genes and subsequently characterized the function of some of these conserved elements using reporter assays in the parasite [18]. Behnke *et al.* (2010) used *T. gondii* tachyzoite gene expression profiles to predict regulatory elements in their upstream sequences [19]. Guo and Silva (2008) mined the non-coding sequences in two *Theileria* genomes and predicted the presence of five putative *cis*-regulatory elements [20]. Two previous studies characterized putative regulatory elements in upstream sequences in *C. parvum*. They grouped genes based on function and looked for conserved DNA motifs in the promoter regions, then correlated these conserved motifs with the RT-PCR expression profiles of the genes examined [21,22]. Many of these classical techniques for the experimental analysis of promoters and gene expression are not feasible in *C. parvum.* Alternate approaches are required. The availability of several genome sequences [23,24] enabled the design of primers and the quantification of expression for each gene using semi-quantitative-RT-PCR [25]. These transcriptome data lay a foundation for inference of gene regulatory mechanisms since they can be used in conjunction with the genome sequence to identify putative *cis*-acting promoter elements.

We utilize expression profiles from a study that generated whole genome expression data for *C. parvum* using semi-quantitative RealTime-PCR of RNA from seven post-infection time points [25]. Out of 3,805 annotated protein-encoding genes, expression data were generated for 3,281. We standardized these data and clustered gene expression profiles using fuzzy *c*-means (FCM) clustering. We identified groups of genes with similar expression patterns throughout the first 72 hours of the intracellular life cycle in HCT-8 epithelial cell culture. We used motif-finding algorithms to identify conserved, overrepresented DNA motifs in the upstream region of genes with very similar expression profiles. A recombinant *C. parvum* DNA-binding protein domain from a putative ApiAP2 transcription factor [CryptoDB: cgd8_810] was generated and tested on PBMs to determine its binding specificity. We demonstrate that cgd8_810 can putatively bind an overrepresented G-box motif, providing support for our methods and potentially implicating this ApiAP2 protein in the regulation of many gene clusters. We additionally investigate *Cryptosporidium*-specific functionally related genes (*Cryptosporidium* oocyst wall proteins), genes found to be co-regulated in other organisms (ribosomal proteins), or genes related by peak expression (72 hours post-infection). We find that each of these groups of genes often appear in the same or similar clusters and share conserved upstream motifs, providing further support for the biological relevance of the identified motifs.

## Results

### Real Time PCR gene expression data

**Figure 1:**
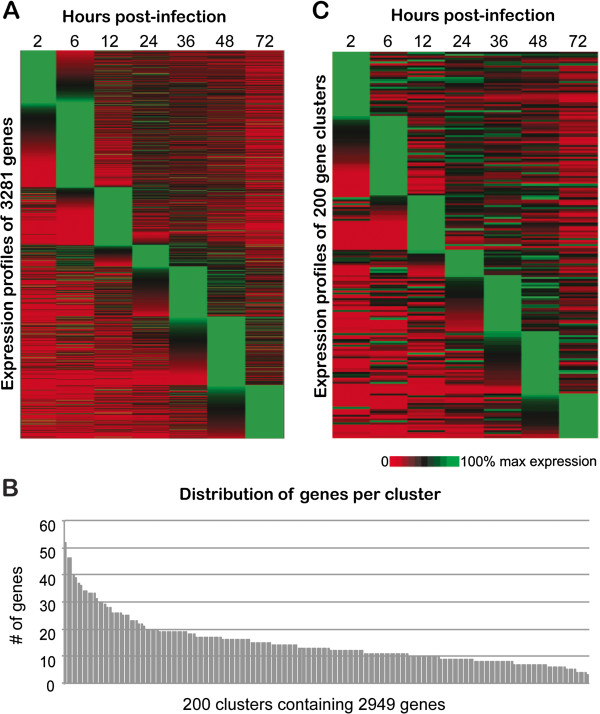
***In vitro C. parvum *gene expression 0-72hr post-infection. A.** Expression profiles of the 3,281 genes used in our study were sorted according to peak expression at each time point. Each row represents the expression profile of a single gene at 2, 6, 12, 24, 36, 48 and 72 hr post-infection. **B.** Distribution of genes per cluster. Of the 3,281 genes used in this study, we were able to cluster 2,949 into 200 clusters. Clusters range in size from 3 to 52 genes, with an average of 14.7 genes and a median of 13 genes per cluster. **C.** Expression profiles of a representative gene from each of the 200 clusters identified using FCM analysis. Each of the 200 rows in the heat map represents a single cluster. Genes were sorted according to peak expression at each time point.

Normalized relative transcript abundance data for 3,281 genes (data from [25]) were standardized as described in Materials and Methods. Expression profiles for all 3,281 genes were sorted according to peak expression at each time point (Figure 1A). There is a cascade of tightly regulated expression across the 72-hour intracellular life cycle of *C. parvum*.

### Identification of co-expressed genes using cluster analysis

The underlying assumption of putative *cis*-regulatory element discovery is that many co-expressed genes (genes that have highly similar expression profiles) are likely controlled by common regulatory elements. In order to identify tightly clustered groups of co-expressed genes, two clustering algorithms, HOPACH and FCM, were implemented using the normalized and standardized semi-quantitative real time PCR expression data. To identify putative *cis*-regulatory elements for these clusters, we searched the upstream regions of all genes in a group/cluster for conserved, overrepresented sequence motifs. One of the major challenges in cluster analysis is determination of the number of clusters present in a given dataset. Most clustering methods are restricted to a one-to-one mapping scheme where one gene is assigned to only a single cluster, known as hard clustering (examples are *k*-means, Self Organizing Maps (SOM) and hierarchical clustering), while soft clustering (such as FCM) can assign genes with a metrics (membership) value indicating the strength of its association with a cluster (see Materials & Methods). Moreover, it is important to have tight clusters of gene profiles that are strongly associated with each other to be most informative for the identification of putative *cis*-regulatory elements. The FCM “fuzzification” parameter, *m*, determines the influence of noise (genes that do not tightly fit the expression pattern of the cluster) on the cluster analysis. For *m*=1, FCM will be equivalent to *k*-means clustering. Increasing *m* reduces the influence of genes with low membership values, which are most likely those genes that are only loosely associated with a cluster. One can assess the stability of clusters by tracking the variation of membership values as *m* and cluster number are increased. Considering inherent biological properties of gene expression as well as the importance of identifying tight and stable clusters, soft clustering using FCM is the most appropriate method for this study.

#### Determination of the optimal FCM parameter set

Estimation of the appropriate values for the two major parameters, *c* and *m*, is crucial to identifying appropriate clusters (see Materials and Methods). Our initial effort to determine the optimal number of clusters using HOPACH cluster analysis resulted in 207 main clusters, of which 124 clusters contained more than two genes (data not shown). Results of additional FCM clustering by increasing *c* and *m* are also shown in Additional file 1: Table S1. For all analyses with minimal *m, m*=1.05, almost all genes were included in the constructed clusters, particularly for *c*=150, 200 and 250. This is equivalent to hard clustering, and false positives in clusters are more likely. The highest membership values were obtained for the analysis with *m*=1.05 and increasing values of *c*, where there were corresponding increments in the overall membership values. As *m* was increased, the number of genes included in clusters decreased (any genes with membership values < 0.5 were excluded). There is also a gradual reduction in the overall average of the membership value for each FCM analysis as *m* increases, indicating fuzzification influences the membership values of genes, and genes with highly similar profiles that form stable clusters will be least affected as *m* is increased. For smaller *c* values, there were larger cluster sizes, but as *c* was increased those main clusters split into smaller clusters (sub-clusters). An ideal parameter set allows sufficient fuzzification while also including an optimal number of genes in the analysis. By tracking the number of genes included in clusters and the range of cluster sizes for each of the FCM cluster analyses (Additional file 1: Table S1), we estimated the ideal parameter set would be one of the four combinations of *m* = 1.15 or 1.25, and *c* = 150 or 200. In order to fix the optimal parameter set, we looked for the significant presence of the core motifs of three previously predicted *C. parvum cis*-regulatory elements [21,22] in the upstream sequences of the genes clustered by the four possible FCM analyses. We performed MEME analysis on the upstream sequences of all clusters (150 and 200) and tracked the number of clusters with significant presence of the three core motifs (5′-GCATGC-3′ and 5′-GGCGGG-3′, both previously reported overrepresented upstream of a subset of glycolysis genes [22]; and 5′-GGGGGG-3′, previously reported overrepresented upstream of 11/12 *C. parvum* heat shock genes [21]). The parameter set *m*=1.25 &*c*=200 produced the most clusters wherein all three core motifs were conserved and overrepresented in upstream regions relative to other FCM parameter combinations. *C. parvum* has 3,805 annotated protein-encoding genes. Using this final parameter set, we were able to cluster 2,949 of the 3,281 genes for which we had expression data, or 77.5% of the genome, into the 200 clusters (Additional file 2: Figure S8). Cluster sizes range from 3 to 52 genes (average = 14.7, median = 13; Figure 1B), with the majority of clusters (107) having between 10 and 19 genes.

All 200 expression profiles generated using FCM cluster analysis were sorted by peak expression at each time point and are displayed in heatmap format, where each row represents a cluster (Figure 1C). Representative expression profiles for each of these clusters closely recapitulate the tightly regulated expression cascade of all 3,281 genes across the 72-hour intracellular life cycle of *C. parvum* (Figure 1A), with some differences; gene expression patterns can be more easily discerned among the 200 clusters, particularly those with multiple peaks in expression. Gene IDs for all genes associated with each cluster can be found in Additional file 1: Table S2. Seventy-four clusters showed at least one biological process GO term enrichment based on the hypergeometric statistical test. Not all genes have predicted GO terms, which explains the limited number of clusters with significant GO term enrichment. This reflects the lack of available experimental data in *C. parvum* relative to other apicomplexan parasites. We predicted at least one conserved and significantly over represented DNA motif in the upstream regions of genes in 198 of 200 clusters.

### Determination of a putative transcription factor binding site

Two N-terminal GST-tagged ApiAP2 protein domains (the previously tested cgd2_3490 [CryptoDB: cgd2_3490] [26] used as a control, and the putative cgd8_810) were produced as described previously [10] and tested on protein-binding microarrays to determine their binding specificities. Protein-binding microarrays, composed of chips dotted with all possible double-stranded DNA 10-mers, are able to determine transcription factor binding specificity with great accuracy, with results comparable to *in vivo*-determined binding specificities [14]. As previously documented, the ApiAP2 domain cgd2_3490 binds the palindromic site 5′-[T/C]GCATGC[A/G]-3′, confirming our methods. Our predicted ApiAP2 cgd8_810 binds the motif 5′-G.GGGG-3′, referred to as the G-box, which is discussed in detail below. These data represent the second experimentally determined putative transcription factor binding preference for a *C. parvum* protein.

### Conserved DNA sequence motifs and their possible biological relevance

Using three *de novo* pattern-finding algorithms, MEME, AlignACE and FIRE, we mined the upstream region of all genes present in each of the 200 identified clusters. Twenty-five statistically significant conserved motifs were identified by at least one of the three algorithms (Table 1; Additional file 1: Table S3). All three pattern-finding algorithms identified motifs 1, 2 and 3, while only MEME and AlignACE identified motifs 4, 5 and 6. Motifs 7 to 25 were identified by FIRE alone. In the case where multiple algorithms identified a motif, MEME counts of genes and clusters possessing the motif are used for the purpose of presentation. Motif identification statistics from all algorithms are reported in Additional file 1: Table S3.

**Table 1:** List of 25 overrepresented motifs identified in this study

**Motif family**	**Motif number**	**Consensus motif pattern**	**Algorithms that identified the motifs**			**No. of clusters whose upstream sequences showed significant overrepresentation**
		5′ -> 3′	MEME	AlignACE	FIRE	
**AP2_1-like**	Motif 1	BGCATGCAH	+	+	+	33
	Motif 7	ACATGY	-	-	+	6
	Motif 8	HTGCACH	-	-	+	10
	Motif 11	MAMTGCA	-	-	+	4
	Motif 23	DRMTTSCATB	-	-	+	2
**G-box-like**	Motif 2	DTGTGGGG	+	+	+	38
	Motif 6	KKGRGGGGRR	+	+	-	16
**E2F-like**	Motif 3	DTTGSCGCCH	+	+	+	114
	Motif 4	TTTGGCGGGAAV	+	+	-	47
**GAGA-like**	Motif 5	GDGRRRRARARRRARA	+	+	-	12
	Motif 13	WATTGCA	-	-	+	6
**CAAT-box-like**	Motif 16	TTTTGCM	-	-	+	7
	Motif 20	BTAKTGCD	-	-	+	8
	Motif 10	RMGACG	-	-	+	1
**Unknown set 1**	Motif 12	GAGWCA	-	-	+	5
	Motif 15	GAYCTMD	-	-	+	9
	Motif 17	VYGTCBC	-	-	+	1
	Motif 18	WTAGACR	-	-	+	1
	Motif 19	HTAGVTCW	-	-	+	1
	Motif 9	YTTACAT	-	-	+	12
**Unknown set 2**	Motif 24	KATYTRCAH	-	-	+	3
**Other unknown**	Motif 14	MAACTA	-	-	+	122
	Motif 21	VRTRAGGAD	-	-	+	3
	Motif 22	HTKWYGAC	-	-	+	5
	Motif 25	WMTAANGA	-	-	+	12

IUPAC codes are used to represent each motif. The algorithm(s) that identified each motif are indicated, as well as the number of clusters containing the overrepresented upstream motif.

#### Overrepresented motif families

**Figure 2:**
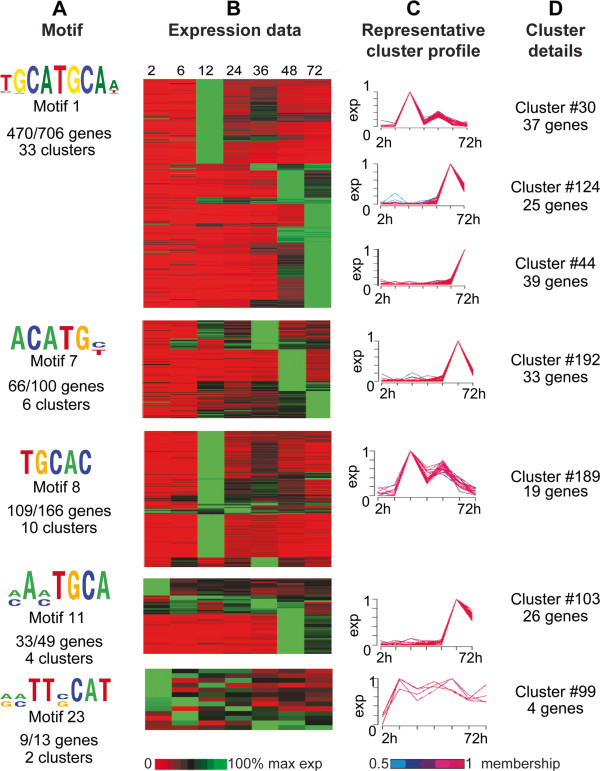
**Data supporting identification of AP2_1-like motifs. A.** AP2_1-like motifs. Motif name and total number of genes possessing each motif per total genes in all clusters where the motif is overrepresented are indicated. **B.** Expression data for 2, 6, 12, 24, 36, 48 and 72 hours post-infection for all genes from each cluster where the motif is overrepresented. Each row indicates a gene; rows are sorted first by cluster, then by peak expression at each time point. Gene IDs for genes associated with each cluster can be found in Additional file 1: Table S2. Expression is indicated on a scale of 0-100% of max for each gene. **C.** Seven representative cluster profiles selected from the 55 clusters containing overrepresented AP2_1-like motifs. Line colors for individual gene profiles indicate the membership values of that gene profile to the cluster ranging from 0.5 to 1. Each cluster profile is located next to the corresponding rows in the gene expression heatmap. **D.** Cluster number and total number of genes in each displayed representative cluster.

We further grouped the 25 identified motifs into 11 motif families based on sequence similarity (as determined via the STAMP tool [27] at an e-value of 1e-3 or better; Table 1). Motifs 1, 7, 8, 11 and 23 are highly similar to the palindromic ApiAP2 binding site 5′-GCATGCA-3′, a well-documented motif in Apicomplexa. We have designated it “AP2_1”. The AP2_1 motif was previously noted to be overrepresented in the non-coding regions of *C. parvum* in a study of chromosome 6 [28]. It was also previously identified as a potential *cis*-regulatory element in *C. parvum*[22] in the upstream sequences of a subset of glycolysis pathway genes. De Silva *et al.* (2008) showed that orthologous ApiAP2 proteins from *P. falciparum* [PlasmoDB: PF14_0633; New ID PF3D7_1466400] and *C. parvum* [CryptoDB: cgd2_3490] both bind the 5′-TGCATGCA-3′ core sequence [26]. The AP2_1 motif is known to be enriched upstream of *P. falciparum* sporozoite-specific genes, which suggested a role in sporozoite-specific transcriptional regulation [16]. Yuda *et al*. (2010) subsequently proved that the *Plasmodium berghei* ortholog of ApiAP2 PF14_0633 [PlasmoDB: PBANKA_132980] binds the AP2_1 motif and is essential for regulation of sporozoite-specific genes [11]. Outside the *Plasmodia*, this motif is also overrepresented in the non-coding regions of other apicomplexan parasites, including *T. gondii* (TRP-2 motif) [18] and *E. tenella*[28]. In this study, 55 clusters of co-expressed genes (corresponding to 1,034 genes) were predicted to have statistically significant overrepresentation of the AP2_1 motif in the upstream regions of their genes (Figure 2A-D). The majority of these clusters have lower levels of expression at 2, 6, and 24 hr post-infection. We investigated the possible biological relevance of these gene clusters using hypergeometric tests for biological process GO term enrichment. Glycolysis, cellular polysaccharide metabolic process, carbohydrate metabolism, post-translational protein modification, protein phosphorylation and regulation of biological quality are all significantly enriched.

**Figure 3:**
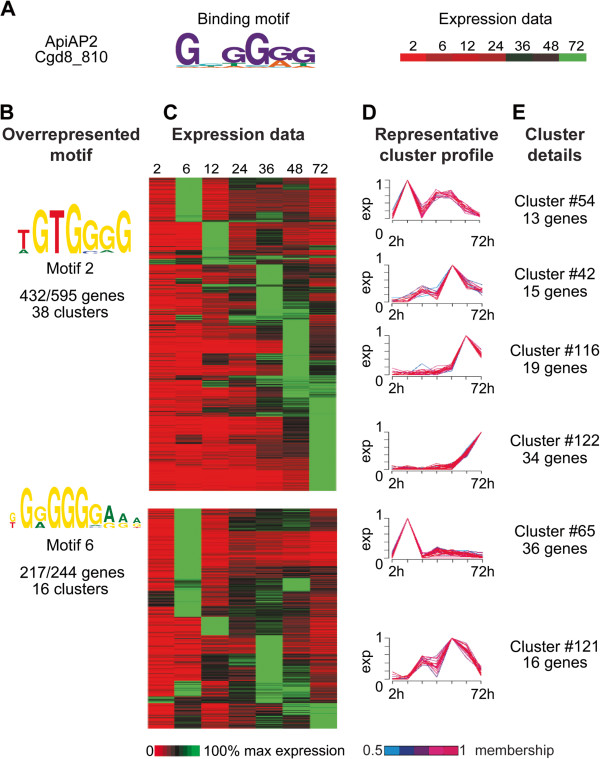
**Data supporting identification of a G-box-binding ApiAP2 and G-box-like motifs. A.** Binding motif for ApiAP2 domain Cgd8_810 as determined by protein-binding microarray. Cgd8_810 expression data for 2, 6, 12, 24, 36, 48 and 72 hours post-infection are indicated. **B.** Identified G-box-like motifs overrepresented in cluster upstream regions. Motif name and total number of genes possessing each motif per total genes in all clusters where the motif is overrepresented are indicated. **C.** Expression data for all genes from each cluster where the motif is overrepresented. Each row indicates a gene; rows are sorted first by cluster, then by peak expression at each time point. Gene IDs for genes associated with each cluster can be found in Additional file 1: Table S2. Expression is indicated on a scale of 0-100% of max for each gene. **D.** Six representative cluster profiles selected from the 54 clusters containing overrepresented G-box-like motifs. Line colors for individual gene profiles indicate the membership values of that gene profile to the cluster ranging from 0.5 to 1. Each cluster profile is located next to the corresponding rows in the gene expression heatmap. **E.** Cluster number and total number of genes in each displayed representative cluster.

Motifs 2 and 6 (5′-G[T/G/A]GGGG-3′) are very similar to the G-box motif previously reported in *C. parvum* in the upstream region of a subset of genes involved in DNA metabolism, as well as 8/18 *P. falciparum* heat shock genes and 11/12 *C. parvum* heat shock genes [15,22]. Expression profiles were available for 11 out of the 12 *C. parvum* heat shock genes, and they grouped into nine different clusters. The G-box motif was significantly overrepresented upstream of the genes in only two of those clusters (totaling 43 genes). Promoter regions of the genes contained in the remaining clusters contained G-box motifs, but their presence was not statistically significant within their respective clusters. PBM results for putative *C. parvum* ApiAP2 transcription factor cgd8_810 indicate it binds the G-box motif. G-box-like motifs are overrepresented in the upstream sequences of 54 *C. parvum* gene expression clusters (corresponding to 839 genes) (Table 1; Figure 3A-D), and again we note that these clusters are for the most part not active 2 hr post-infection. Some of the GO terms enriched in these gene clusters are DNA packaging, nucleosome organization, organophosphate metabolic process, alcohol metabolic process, mRNA metabolic process, ubiquitin-dependent protein catabolic process, phospholipid biosynthetic process, membrane lipid biosynthetic process and DNA metabolism. Of the 54 clusters containing G-box-like motifs in their upstream sequences, 16 clusters also have AP2_1-like motifs, suggesting the possibility of joint involvement in regulation of these genes (Additional file 1: Table S4).

**Figure 4:**
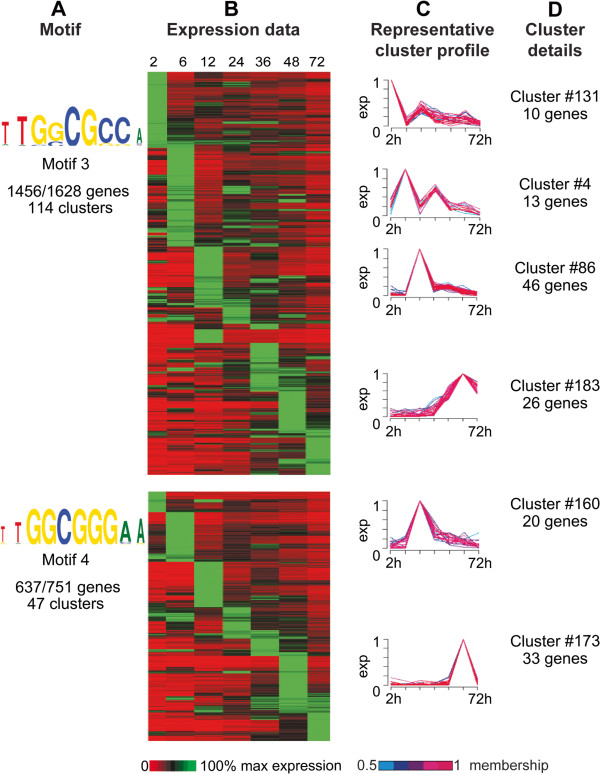
**Data supporting identification of E2F-like motifs. A.** E2F-like motifs. Motif name and total number of genes possessing each motif per total genes in all clusters where the motif is overrepresented are indicated. **B.** Expression data for 2, 6, 12, 24, 36, 48 and 72 hours post-infection for all genes from each cluster where the motif is overrepresented. Each row indicates a gene; rows are sorted first by cluster, then by peak expression at each time point. Gene IDs for genes associated with each cluster can be found in Additional file 1: Table S2. Expression is indicated on a scale of 0-100% of max for each gene. **C.** Six representative cluster profiles selected from the 161 clusters containing overrepresented E2F-like motifs. Line colors for individual gene profiles indicate the membership values of that gene profile to the cluster ranging from 0.5 to 1. Each cluster profile is located next to the corresponding rows in the gene expression heatmap. **D.** Cluster number and total number of genes in each displayed representative cluster.

Motif 3 (core sequence pattern 5′-[C/G]GCGC[G/C]-3′) and motif 4 (core sequence pattern 5′-GGCGGG-3′) are highly similar to the binding site of the E2F-DP transcription factor, which represents an important class of TFs that function as major regulators of the cell cycle and apoptosis [29]. E2F transcription factors have been studied extensively in a broad range of organisms, such as mammals [30], worm [31], frog [32], fly [33] and plants [34]. The E2F family is comprised of two subfamilies: E2F and DP. One member of each subgroup partners to form a heterodimeric complex that binds to the promoter of a multitude of target genes. The E2F motif was previously noted to be overrepresented in the non-coding regions of *C. parvum* chromosome 6 [28], though it was not identified as an E2F motif. The typical conserved sequence of the E2F/DP binding site is 12 bp in length, which consists of a 6 bp CG core flanked by T- and A-enriched sequence. This conserved central CG motif ([C/G]GCGC[G/C]) is symmetric, and amino acids that contact these bases are conserved amongst all known E2F and DP proteins [29]. Ramirez-Parra *et al.* (2003) found that consensus motifs 5′-TTTCCCGCC-3′ and 5′-TTTGGCGGG-3′ are the most abundant motifs in the *Arabidopsis* genome, and these sites were previously shown to be able to direct binding of E2F/DP [35]. In *C*. *parvum*, Templeton *et al*. (2004) reported the existence of two E2F/DP winged-helix DNA-binding domain transcription factor pairs not found in *P*. *falciparum*[36,37]. However, the specific roles these transcription factors play in *C*. *parvum* are unknown. In fly, worm and mammals, E2F transcription factors have been shown to form complexes with members of the retinoblastoma protein family (pRb) as well as MYB and other proteins to regulate cell cycle progression (reviewed in [38]). pRb acts as a repressor of E2F-directed cell proliferation; pRb has been found to be inactivated in many cancers (reviewed in [39]). No *C*. *parvum* (or any other apicomplexan) orthologs to pRB proteins and most other protein components of the complex are contained in the OrthoMCL database (orthomcl.org), with the exception of *D*. *melanogaster* RPD3 and *C*. *elegans* LIN-53. E2F motifs are overrepresented upstream of genes present in 163/200 clusters (corresponding to 2,379 genes), making E2F-like motifs the most abundant putative transcription factor binding sites in *C*. *parvum* (Figure 4A-D). Clusters containing overrepresented E2F-like motifs in their upstream regions do not show any particular expression patterns and genes with peak expression can be observed at all examined time points. *C*. *parvum* possesses three putative E2F transcription factors and two DP1 binding partners (Table 2). Expression data is available for two of the three E2F transcription factors and both DP1 binding partners, and all are maximally expressed at 2 and 12 hours post-infection, though they are expressed at some level at all time points [25]. Of the 20 clusters containing overrepresented E2F motifs maximally expressed at 2 hours, 45% have E2F as the only overrepresented upstream motif. This finding suggests that E2F regulation could be sufficient to drive expression of this subset of clusters. As described in the materials and methods, GO enrichment analysis revealed that clusters having overrepresented E2F motifs are statistically enriched for a number of biological processes, including structure-specific DNA binding, gene expression, translation, DNA metabolic process, response to DNA damage stimulus, DNA repair, regulation of nucleobase, nucleoside, nucleotide and nucleic acid metabolic process, RNA processing, RNA binding, ribonucleoprotein assembly, nucleocytoplasmic transport, golgi vesicle transport, cell redox homeostasis, establishment of protein localization to lipids, secretion by cell, lipid transport, carbohydrate transport and glycolysis. E2F-like motifs have previously been found to be overrepresented in *C*. *parvum* at the promoter regions of subsets of genes associated with DNA replication and glycolysis [22].

**Table 2:** **Possible *C parvum *transcription factors**

**Domain**	**# of *C. parvum *proteins**
**ApiAP2**	19
**E2F/TDP**	2/3
**MYB**	9*
**Zinc finger**	
GATA DNA-binding	3
C_2_H_2_	27*
**bZIP**	
CCAAT-binding	3
other	1

Domains commonly associated with transcription factors and their counts in *C. parvum* as determined by text searches at CryptoDB.org. ApiAP2 protein counts determined using custom-built HMMs. *Presence of several of these domains, particularly the C2H2 Zinc finger and Myb, do not necessarily indicate the protein acts as a transcription factor.

**Figure 5:**
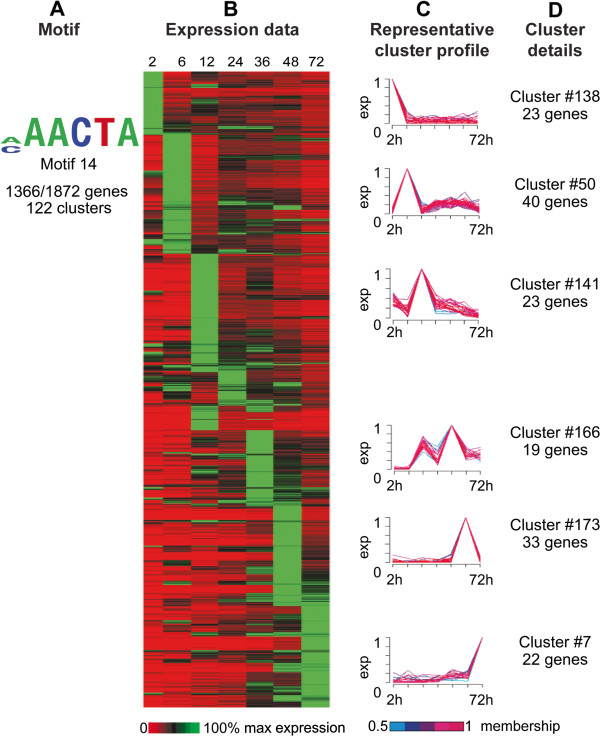
**Data supporting identification of Unknown motif 14. A.** Unknown motif 14. Total number of genes possessing the motif per total genes in all clusters where the motif is overrepresented is indicated. **B.** Expression data for 2, 6, 12, 24, 36, 48 and 72 hours post-infection for all genes from each cluster where the motif is overrepresented. Each row indicates a gene; rows are sorted first by cluster, then by peak expression at each time point. Gene IDs for genes associated with each cluster can be found in Additional file 1: Table S2. Expression is indicated on a scale of 0-100% of max for each gene. **C.** Six representative cluster profiles selected from the 122 clusters containing overrepresented Unknown motif 14. Line colors for individual gene profiles indicate the membership values of that gene profile to the cluster ranging from 0.5 to 1. Each cluster profile is located next to the corresponding rows in the gene expression heatmap. **D.** Cluster number and total number of genes in each displayed representative cluster.

Motif 14, with the A-rich core 5′-[A/C]AACTA-3′, is the second-most overrepresented motif in the upstream regions of the genome, found upstream of 1,366 of the 1,872 genes in 122 of 200 clusters. It does not have significant similarity to known regulatory motifs. These clusters are maximally expressed at any of the seven time points (Figure 5A-D). This motif appears in conjunction with many different motifs upstream of clusters with very different expression profiles (Additional file 1: Table S4). Unknown Motif 14 can occur on either strand, at any coordinate in the upstream region, anywhere from one to eight times per upstream region. The ubiquity of this motif and the wide variation between combinations of motifs and expression profiles makes it very difficult to attribute any particular expression pattern to motif 14.

The remaining 11 motifs fall into various families that do not appear to be significantly similar to known regulatory motifs and are discussed in Additional file 2.

### Evidence for biological relevance of select clusters and motifs

#### Ribosomal proteins

Identification of overrepresented motifs upstream of genes that cluster by expression profile gives us a global view of potentially co-regulated genes. To investigate potentially co-regulated genes on a more targeted scale (i.e. not necessarily computationally clustered), we examined several groups of functionally-related genes or genes expressed at a specific point in time, starting with ribosomal proteins. We examined expression data for 68 of *C*. *parvum*’s 81 predicted ribosomal proteins (all of the *C*. *parvum* ribosomal proteins for which we have expression data) and compared them to 68 *P*. *falciparum* ribosomal proteins expressed during the intraerythrocytic cycle (*P*.*f*. data from [40]). Sixty of 68 *C*. *parvum* ribosomal proteins clustered into 22 groups; eight had expression profiles too dissimilar to be clustered. Sixty-three percent of clustered ribosomal proteins fall into five clusters (cluster #4, four ribosomal proteins; #6, 13 ribosomal proteins; #20, five ribosomal proteins; #35, 11 ribosomal proteins; and #91, five ribosomal proteins). The majority of ribosomal proteins have a bimodal expression pattern, peaking at both 6 and (to a lesser, more variable extent) 24 hours, corresponding to stages in the life cycle thought to be translationally active in the production of trophozoites and type 1 merozoites [25]. Ribosomal proteins have been documented to be tightly co-regulated in other organisms such as yeast [41] and to be stage-specifically regulated in the apicomplexan *Eimeria tenella*[42]. Though we see more variability in ribosomal protein expression in *Cryptosporidium* in terms of the number of clusters, expression of these proteins still appears in clusters.

**Figure 6:**
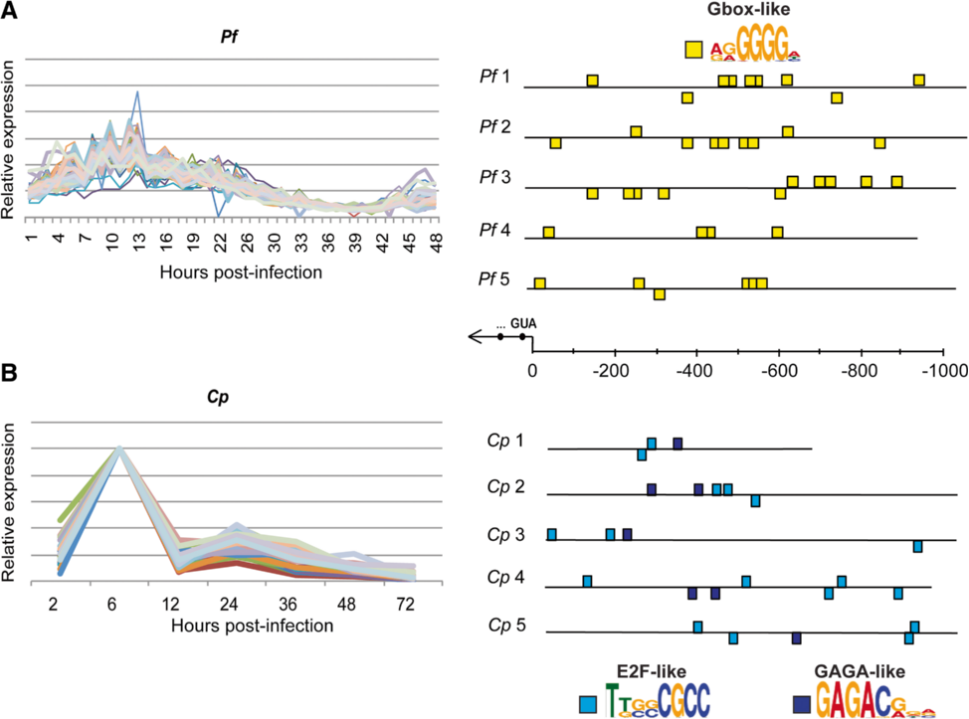
**Overrepresented motifs upstream of ribosomal protein genes in *P. falciparum *and *C. parvum*. A.** Expression profiles for 68 *P. falciparum* (*Pf*) co-expressed IDC ribosomal proteins (data from Bozdech *et al.* 2003). **B.** Expression profiles for 25 *C. parvum* (*Cp*) co-expressed ribosomal proteins from clusters #6, #20 and #35. Five representative upstream regions are shown for each organism out of 68 *Pf* and 60 *Cp* respectively. Upstream regions for each of these genes were mined for overrepresented motifs (see Materials and Methods). As previously documented, the upstream regions of *Pf* ribosomal proteins contain overrepresented G-box motifs (Essien and Stoeckert, 2010). *Cp* ribosomal proteins have E2F-like and GAGA-like motifs overrepresented upstream.

Upstream regions of 68 co-expressed *P. falciparum* ribosomal protein genes (as identified in [21]) as well as 60 clustered *C. parvum* ribosomal protein genes were mined for overrepresented motifs using MEME. We confirm the presence of the G-box motif that was previously noted upstream of *P. falciparum* ribosomal proteins (Figure 6A) [43]. Upstream sequence analysis of this subset of *C. parvum* ribosomal proteins indicates that E2F-like and GAGA-like motifs are overrepresented (Figure 6B). Campbell *et al*. (2010) identified the G-box binding ApiAP2 transcription factor PF13_0235 as the putative regulator of *P. falciparum* ribosomal proteins [13], noting that the mRNA expression profiles of this protein correlated very tightly with ribosomal protein expression. The G-box motif is also conserved upstream of three other *Plasmodium* species’ ribosomal genes, as well as piroplasm ribosomal genes [43]. The putative E2F transcription factor expression profiles do not closely correlate with the expression of these *C. parvum* ribosomal proteins, though E2Fs are expressed at some level at all time points. There are no predicted *trans* factors for the GAGA-like motif in *C. parvum*.

#### Cryptosporidium Oocyst Wall Proteins (COWPs)

**Figure 7:**
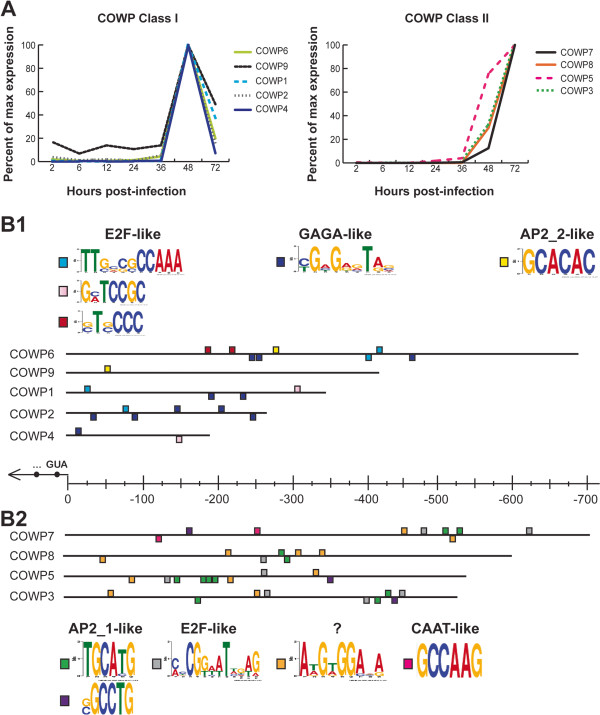
**Overrepresented motifs upstream of COWPs by subclass. A.** Expression profiles of Class I and Class II COWPs. The five COWPs that fall into Class 1 peak at 48 hrs post-infection and then decline. The remaining four Class II COWPs begin rising at 48 hrs and peak at 72 hrs. **B1.** The upstream regions of each of the Class I COWPs contain five overrepresented motifs that fall into three groups. Upstream regions for each of these genes were mined for overrepresented motifs (see Materials and Methods). Three motifs overrepresented upstream of Class I COWPs are closely related to E2F binding sites. A GAGA-like motif and an ApiAP2 motif identified in *P. falciparum* (Campbell *et al.* 2010; here we designate this motif AP2_2) are also overrepresented upstream of Class I COWPs. **B2.** The upstream regions of each of the Class II COWPs contain five overrepresented motifs. Two motifs are similar to a documented ApiAP2 binding site across apicomplexans. E2F-like and CCAAT-box-like motifs are also overrepresented. The remaining motif is unknown and does not appear related to any of the 25 motifs identified in this study.

COWP genes have two distinct expression profiles: four COWP genes have peak expression at 48 hours with a decline at 72 hours, which we have termed Class I; and five genes with expression increasing steadily from 36 hours to peak at 72 hours, Class II (Figure 7A). Though subclasses of COWPs have not been previously described, the expression data utilized in this study generally agree with what has previously been shown for COWPs [44] with the exception of COWP1 and COWP6, which both belong to Class I according to the Mauzy dataset [25] but belong to Class II according to the Templeton dataset [44]. Three E2F-like motifs, one GAGA-like motif and one motif with the consensus 5′-GCACAC-3′, similar to several *P. falciparum* ApiAP2 binding sites as well as the binding site for a recently characterized *T. gondii* ApiAP2 which acts as a repressor [45] are overrepresented upstream of Class I COWP genes (Figure 7B1). We have designated 5′-GCACAC-3′ as AP2_2. Class II COWP genes share the E2F motif but otherwise have very different motifs: AP2_1-like motifs, a CCAAT-box-like motif, and an unknown motif with the consensus 5′-A[T/A]G[T/A]GGA.A-3′ which is not similar to any of our 11 overrepresented motif families (Figure 7B2). Mass spectroscopy data has also indicated five other possible oocyst wall proteins (“POWPs”) present in trace amounts in excysted, purified oocyst walls [46]. Expression profiles for these five proteins also fall into our two proposed classes, with POWP2, POWP4 and POWP5 falling into Class I, and POWP1 and POWP3 falling into Class II (data not shown).

#### Transcripts peaking at 72 hours post-infection

**Figure 8:**
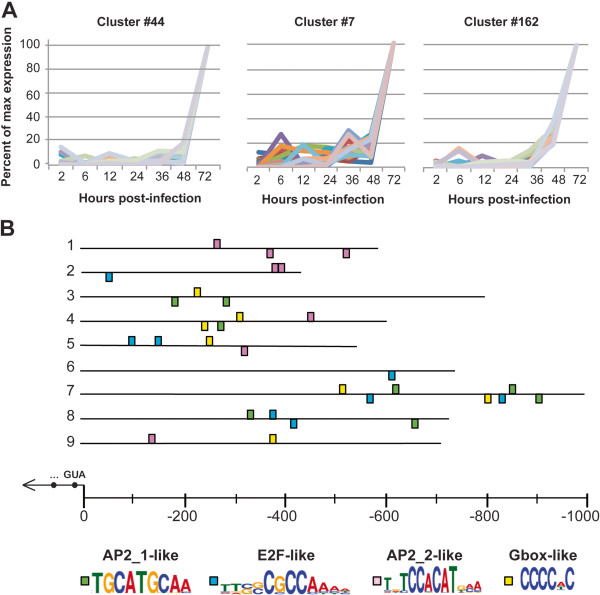
**Overrepresented motifs upstream of genes in clusters peaking primarily at 72 hrs post-infection. A.** Clusters peaking primarily at 72 hrs post-infection. **B.** Overrepresented motifs upstream of genes in these clusters. Nine representative upstream regions are shown out of 105 searched. Upstream regions for each of these genes were mined for overrepresented motifs (see Materials and Methods). The upstream regions of genes in clusters peaking primarily at 72 hours share four overrepresented motifs. Two of these motifs are similar to previously identified ApiAP2 binding sites. One binding site is E2F-like. The remaining site is similar to the G-box noted in other apicomplexans, which we have demonstrated is an ApiAP2 binding site in *C. parvum*.

*C. parvum in vitro* parasite growth fails somewhere from 72 to 96 hours post-infection. Following completion of the formation of type 1 meronts at 24–36 hours and the release and reinvasion of type 1 meronts into new cells, development can occur along two pathways: An asexual round of replication can lead to more type 1 meronts, or some parasites will form type 2 merozoites that upon release (72–96 hours) will form the sexual stages of the parasite. Type 1 and type 2 merozoites are morphologically indistinguishable by light microscopy [25]. While gametocytes are occasionally seen in *in vitro* culture, oocysts are never observed and parasite development stops. We examined the three clusters which peaked only at 72 hours (Figure 8A) to examine what they may indicate about parasite biology at this critical time point (clusters #44, #7, and #162 comprising 22, 40, and 46 genes, respectively). No GO-terms are over-enriched for genes in clusters 44 or 7, though genes involved in proteolysis and carbohydrate metabolic processes are over-enriched in cluster 162. AP2_1-like, E2F-like, AP2_2-like and G-box-like motifs are over-enriched upstream of genes in these three clusters (Figure 8B). ApiAP2 gene cgd2_3490, which is AP2_1-binding, is maximally expressed at 72 hours post-infection, as is the G-box-binding ApiAP2 cgd8_810. No AP2_2-like binding proteins have been identified in *C. parvum*, but it is reasonable to believe that ApiAP2s orthologous to the CACACA-binding ApiAP2s in *P. falciparum* could also bind this motif, given the conservation of binding sites found between another *P. falciparum*/*C. parvum* ApiAP2 ortholog pair [26]. The finding that 3/4 of the overrepresented motifs upstream of these late-peaking genes are potential ApiAP2 binding sites suggests that ApiAP2 proteins are important regulators in the later stages of the parasite’s intracellular life cycle.

## Discussion

Little is known about transcriptional regulation in apicomplexans in general and *Cryptosporidium* in particular, though recent studies in *Plasmodium* and *T. gondii* have begun to reveal the tremendous complexity of transcriptional regulatory mechanisms in these parasites [13,19,26]. In this study, we have used bioinformatics approaches to analyze available *C. parvum* transcriptome data and genome sequence to advance our understanding of possible regulatory mechanisms in this experimentally intractable parasite.

Clustering of gene expression profiles is often used to reveal patterns of gene regulation. Such analyses provide valuable information regarding which genes are expressed at a particular time in the life cycle. Mauzy *et al*. (2012) used the DIANA algorithm available in the “cluster” package in R [47,48] to cluster 3,281 *C. parvum* genes into nine groups based on similarity of expression profile [25]. These large clusters, consisting for the most part of hundreds of genes each, allowed them to observe general functional trends for genes expressed at each stage of the life cycle. Among their findings, they note that transcripts expressed at each time point make biological sense in the context of what is known about *C. parvum* biology at each of the examined life cycle stages. For example, genes involved in protein synthesis and degradation, nutrient availability, and ribosome biogenesis are highly expressed in the trophozoite stage (~6 hours post-infection), where the parasite is growing, absorbing nutrients and preparing for the first round of cell division. While these observations are certainly useful for a global understanding of the *C. parvum* transcriptome and validation of the dataset, the hundreds of genes found in each of these clusters are not likely to be truly co-regulated in the organism, and the entire diversity of *C. parvum* gene expression profiles (Figure 1A) cannot be captured in only nine gene expression clusters.

We have clustered 2,949 *C. parvum* genes into 200 putatively co-regulated clusters based on their expression profiles. Many lines of evidence support the biological relevance of many of these clusters, namely: (1) expression profiles within each cluster are well-correlated, and there are statistically significant overrepresented motifs upstream of the genes comprising 198 of 200 clusters; (2) identified overrepresented motifs fall into 11 motif families, many of which could potentially be bound by known *C. parvum* transcription factors, as well as one previously unknown G-box-binding ApiAP2 transcription factor, cgd8_810; and (3) the two examples of functionally related and known co-expressed genes (COWP genes and ribosomal proteins) are clustered.

Though functionally related genes, or genes known to be co-expressed in other organisms were often clustered together, there are instances where these genes fall into disparate clusters in *C. parvum*. The 11/12 heat shock proteins for which expression data are available grouped into 9 different clusters, yet all share the G-box motif. The G-box is unlikely to be the only motif contributing to regulation of these genes; combinatorial transcriptional regulation, or other mechanisms such as epigenetic regulation prior to transcription may be involved to produce these different expression patterns. The AP2_2 motif is not found overrepresented upstream of any individual cluster, but when we group several clusters (in the case of late-peaking genes) or functionally related genes (in the case of COWP genes) together, this motif is statistically overrepresented. These observations indicate that the 200 clusters we identified may be finer-scale or overly divided relative to larger overall patterns or not indicative of truly co-regulated genes. It may be the case that some of the identified clusters can be collapsed into larger clusters, and that we have overestimated the number of clusters. Our FCM parameter exploration suggests that the true number of clusters is somewhere between 150 and 200, with 200 producing the highest number of clusters with known regulatory elements conserved upstream. Alternatively, it must also be considered that genes have been incorrectly assigned to clusters. As noted in Mauzy *et al*. (2012), *C. parvum* cultures cannot be synchronized beyond the first 24 hours post-infection. Thus RNA collected past this time point is a mix of life cycle stages, and gene expression profiles may begin to vary with unsynchronized parasite development in the culture. Despite this possibility, we find that many clusters still exhibit tight co-expression at later time points (Additional file 2: Figure S8), suggesting that culture asynchrony may not be a big problem. Mauzy *et al*. (2012) noted subsets of genes that are expressed at a single time point only. To investigate the earlier stages of development where culture asynchrony is not an issue, we examined the upstream regions of the 31 genes identified in Mauzy *et al*. as being expressed only at 12 hours post-infection. All 31 genes were found in the same cluster (#170 Figure S8). Upstream motif analysis revealed overrepresented E2F and AP2_1 motifs.

We observe E2F-like and GAGA-like motifs conserved upstream of *C. parvum* ribosomal protein genes. The upstream regions of the ribosomal gene regulon have been examined in several other apicomplexans, and overrepresented motifs in the species examined are largely known ApiAP2 binding sites. *T. gondii* ribosomal proteins were found to have the AP2_1-like motif overrepresented upstream (referred to as TRP-2 in *T. gondii*) [49]; Essien *et al*. also reported conservation of this motif upstream of *N. caninum* ribosomal genes [43]. The ApiAP2 G-box motif is conserved upstream of four out of five examined *Plasmodium* species’ ribosomal genes, as well as piroplasm ribosomal genes [43]. The overrepresentation of different motifs upstream of ribosomal protein regulons across the phylum raises the possibility that there have been multiple transcription factor substitutions in ribosomal protein transcriptional regulation over time. E2F/DP1 transcription factors can be traced back to the last eukaryotic common ancestor [50], making it one of the oldest transcription factor families known, and *Cryptosporidium* is the most basal-branching apicomplexan taxon for which we currently have a genome sequence [51]. It is therefore attractive to consider that E2F regulation of the ribosomal gene regulon is the ancestral state, with switches to, or between, various ApiAP2 transcription factors occurring over the course of apicomplexan evolution. Given the extremely high level of breaks in synteny across the Apicomplexa [52], it is possible to imagine how coding regions can become associated with new and different regulatory regions.

We observe a disparity in the different types of motifs identified by the different algorithms; some motifs were identified by all algorithms, while other motifs were identified by only one or two algorithms. This finding is explained by the differences in these algorithms’ underlying assumptions. MEME and AlignACE discover degenerate motif candidates using an expectation maximization strategy and Gibbs sampling, respectively, from a set of sequences. FIRE uses model-independent mutual information and continuous (e.g., expression log ratios from a single microarray experiment) or discrete (e.g., a clustering partition) data to identify motifs. Due to the theoretical similarity behind the MEME and AlignACE motif discovery methods, there should be a correlation between the motifs identified by them. This is exactly what we observed. The first six motifs (motifs 1 to 6) were identified by both MEME and AlignACE. One of the possible limitations of FIRE is that it may overlook certain highly degenerate motifs, as it initially begins by searching non-degenerate motif representations [17]. Perhaps for these reasons, FIRE did not identify motifs 4, 5 & 6, nor was there a consensus between FIRE and the other two algorithms concerning all clusters identified as having overrepresentations of motifs 1, 2 and 3.

Many of the overrepresented motifs we identified are still of unknown function. Likewise, the binding specificities of most of the putative *C. parvum* transcription factors are not known, particularly the many zinc finger and ApiAP2 proteins; these unknown motifs could represent binding sites for these transcription factor proteins. It is also a possibility that these motifs are not transcription factor binding sites; they might represent some other *cis* element important for other mechanisms of gene regulation, such as binding sites for proteins involved in epigenetic regulation. Alternatively, it is possible that these motifs are not involved in gene regulation and represent some sort of repeat element. Additional studies to elucidate binding sites for the remaining putative *C. parvum* transcription factors and DNA-binding proteins coupled with experiments to determine their binding sites throughout the genome (ie, utilizing ChIP-seq) are needed to distinguish between these possibilities. An additional complication to identifying *cis-*regulatory elements is that *C. parvum* UTRs are largely undefined. Apicomplexan *cis-*regulatory elements have traditionally been identified by looking in the 1-2 kb of sequence directly upstream of coding regions, or until a gene is encountered on either strand [18,22,53]; however, there is some evidence that in highly compacted eukaryotic genomes, such as that of *C. parvum*, transcripts overlap, and UTRs are not necessarily limited to intergenic spaces [54]. The extent of occurrences of overlapping transcripts in *C. parvum* has not been quantified. Thus it is possible that the upstream sequence database we generated, and subsequently the motifs we identified, are not representative of what would be identified in the endogenous promoter. However, the finding that several known *cis*-regulatory elements are overrepresented in our upstream regions lends support that our motif-finding methodology is biologically relevant. Strand-specific RNA-seq data and epigenetic state information for *Cryptosporidium* will reveal the UTR sequences and open chromatin respectively and permit more accurate identification of endogenous promoters.

We also note that the E2F motif is particularly overrepresented throughout the upstream regions of the *C. parvum* genome. This is very interesting, given the absence of E2F/DP1 transcription factor proteins in other apicomplexans. It is an intriguing possibility that *C. parvum* is unusually reliant on a small number of E2F transcription factors for transcriptional regulation. Most of the E2F-interacting proteins important for E2F-mediated transcriptional regulation identified in flies, worms and mammals are absent in *C. parvum*; however a few have been retained (DP1, RPD3, LIN-53). E2F regulatory interactions may be different in *C. parvum* versus other well-studied organisms as a result. Clusters containing overrepresented E2F motifs in the upstream regions of their genes are observed to have maximal expression at any of the seven post-infection time points. The two E2F genes for which we have expression data are expressed at some level at all time points, though E2F cgd1_1570 is maximally expressed at 2 hours post-infection, and E2F cgd6_1430 and both DP1 proteins are maximally expressed at 12 hours post-infection. E2F proteins could thus be available to regulate at all examined time points. However, the presence of the motif does not necessarily indicate that the transcription factor binds it. Indeed, Flueck et al. (2010) recently concluded that *P. falciparum* ApiAP2 protein PFF0200c (which is believed to act as a DNA tethering protein involved in formation and maintenance of heterochromatin, instead of as a transcription factor) only binds instances of its motif that are located in subtelomeric heterochromatin *in vivo*[55]. ChIP-seq experiments to determine whether or not most of these overrepresented motifs act as true E2F binding sites will help to elucidate the importance of E2F transcription factors in *C. parvum* transcriptional regulation.

Our data suggest that in most cases, a single overrepresented motif is not sufficient to explain cluster expression patterns. A notable exception is in the case of E2F motif-containing clusters that peak at 2 hours post-infection, where the E2F motif is the only overrepresented motif detected upstream in 45% of these clusters. Both E2F transcription factors and their DP1 dimerization partners are expressed at this time point and could possibly be driving expression of these clusters. However, peak expression at 2 hours post-infection is not so easily explained, and the presence of the E2F motif is not the only determinant of peak expression at 2 hours post-infection; clusters containing any of our identified overrepresented motifs can peak at this time. Another 45% of E2F-motif-containing clusters also have Unknown motif 14 overrepresented upstream. Unknown motif 14 can occur on either strand, at any coordinate in the upstream region, anywhere from one to eight times per upstream region. Given these variable characteristics and the abundance of Unknown motif 14, it is an attractive possibility that this motif is a general transcription factor binding site; future ChIP-seq experiments, if and when they become technically feasible, will help to determine the function of Unknown motif 14. At this time, the ubiquity of this motif in regions upstream of clusters having a wide variety of expression patterns makes the influence it has on gene expression, if any, very difficult to decipher. We see any manner of combinations of motifs overrepresented upstream of clusters with highly variable expression patterns, which suggests a very complicated interplay between motifs and transcription factors that act together to determine these intricate and precise expression patterns. The variable orientation, spacing, and overall number of overrepresented motifs upstream of clusters all need to be considered to understand *C. parvum* transcriptional regulation.

Functionally related or known co-expressed genes appear together in clusters in the case of ribosomal proteins and COWP genes. Clustering further allowed us to distinguish between two potentially co-regulated classes of COWP: Class I, which peaks at 48 hours, then declines; and Class II, which rises steadily from 36 hours to peak at 72 hours. The E2F binding motif (motif 3) is overrepresented upstream of Class I COWPs, while a known ApiAP2 binding site (motif 1) is overrepresented upstream of Class II. It is possible that this differential regulation indicates functional differences between the two classes of COWP. It should be noted that the expression data for COWPs utilized in our study differ slightly from what has previously been described [44]. The gene membership between Class I and Class II differs slightly between datasets, with COWP1 and COWP6 changing classes. Despite these differences, both datasets suggest two differentially regulated classes of COWPs. Electron microscopy data indicate that the *C. parvum* thick-walled oocyst is divided into three layers: a ~10 nm outer layer, sometimes referred to as the outer veil [46]; a rigid, SDS- and protease-resistant 2.5 nm electron-lucent middle layer that is largely uncharacterized; and a thick, multi-zoned inner layer of 37.4 nm [56]. No mechanism has yet been indicated for how the oocyst wall is formed. Protein localization data indicate that COWP1 (a member of the earlier-expressed class of COWP) localizes to the inner oocyst wall [57]. An antibody to COWP8 (a member of the later-expressed Class II) is only reactive to ruptured oocysts [44], indicating this COWP is not expressed on the oocyst surface, but there is no precise localization data for COWP8. To our knowledge no other COWP protein localization data are available. With these limited data, it is tempting to speculate that the earlier class of COWPs represents components of the inner oocyst membrane, while the later-expressed class of COWPs builds on this earlier structure to help form the remaining layers. Mass spectroscopy data on excysted, purified oocyst walls without the outer veil indicate that COWP1, COWP6 and COWP8 are the most abundant COWPs in these parts of the oocyst wall, with COWP2, COWP3 and COWP4 present in trace amounts. COWP5, COWP7 and COWP9 were not detected at all [46]. Chatterjee et al. also identify five other possible oocyst wall proteins (“POWPs”) present in trace amounts in the mass spec data. Expression profiles for these five proteins also fall into our two proposed classes, with POWP2, POWP4 and POWP5 falling into Class I, and POWP1 and POWP3 falling into Class II. This is valuable information as to the composition of the oocyst wall, though these data do not conclusively indicate protein localization. Future localization studies on the remaining COWPs and all POWPs will help investigate the hypothesis that expression class is somehow related to role in oocyst wall structure.

## Conclusions

Bioinformatic approaches combined with experimental DNA binding site determination for an ApiAP2 protein have allowed us to identify overrepresented upstream sequence motifs that are correlated with clustered gene expression profiles. This information allows us to postulate transcriptional mechanisms in *C. parvum*. We have generated testable hypotheses that will further elucidate regulatory mechanisms and other aspects of *C. parvum* biology.

## Methods

### Gene expression data

We utilized expression data generated for 3,281 of the predicted 3805 *C. parvum* genes (data from [25]). Briefly, HCT8 cell infection was carried out according to [58-60] wherein 2–2.5 × 10^7^ oocysts were added to each culture dish at time (t) = 0 hr and total RNA was collected at 2, 6, 12, 24, 36, 48, and 72 hrs post infection. RNA was isolated and DNase-treated following manufacturer protocol. cDNA synthesis was accomplished using Superscript III cDNA synthesis kits using a modified version of the manufacturer’s protocol. Real Time PCR was performed on the cDNA with 3,302 primer pairs designed to *C. parvum* genes. At least three biological replicates of each gene for each time point were successfully obtained for 3,281 genes.

### Real time PCR (RT-PCR) data standardization

Normalized RT-PCR data were obtained from [25]. Briefly, the relative transcript abundance for each gene at each time point for each replicate was obtained by normalizing the initial fluorescence (IO) values of a gene to 18S rRNA IO values [7,61]. We took the median of the replicate normalized IO values for each gene at each time point in order to get a representative measure of transcript abundance. We standardized this representative normalized IO expression value to the maximum expressed time point for each gene, in a modified ΔΔCt fashion [62,63]. These normalized, standardized transcript data were used for all further analyses.

### Cluster analysis

In order to identify likely groups of co-expressed genes, two clustering algorithms, Hierarchical Ordered Partitioning and Collapsing Hybrid (HOPACH) and FCM clustering methodologies were implemented using the normalized and standardized expression data obtained from real time PCR.

The HOPACH method combines the strengths of both partitioning and agglomerative clustering methods and was implemented using the HOPACH package [64] available from the Bioconductor repository [65]. Euclidean distance was used as the distance metric. The HOPACH algorithm uses the median silhouette (MSS) criteria [66] to automatically determine the main clusters. The main purpose of implementing this clustering procedure was to estimate the number of clusters inherent in the data. FCM, the soft partitioning clustering method, was implemented using the Mfuzz package [67], which is based on the open-source statistical language R and available from the Bioconductor repository. The FCM clustering algorithm requires two main parameters (*c*, the number of clusters, and *m*, the fuzzification parameter) and uses euclidean distance as the distance metric. FCM assigns to each gene expression vector a membership value in the range [0,1] for each of the *c* clusters. The membership value indicates how well the gene expression vector is represented by the cluster to which it is assigned. Large membership values indicate high correlation of the gene expression vector to its cluster center. The FCM algorithm iteratively assigns the gene expression vector to the cluster with the nearest cluster center while minimizing an objective function. The fuzzification parameter, *m*, plays an important role in deriving robust clusters that are not greatly influenced by noise and random artifacts in the data. If *m* is increased, poorly classified gene expression vectors, which have small cluster membership values, contribute less to the calculation of cluster centers. Two other parameters, *e,* the minimal change in the objective function for terminating the clustering process and *T*_*max*_, the maximal number of iterations, are also specified. In this study, we specified the default value for *e* (0.001) and for *T*_*ma*x_ (100,000 iterations).

In order to select the optimal values of *c* and *m*, we used a combination of heuristics as well as a data-driven approach by implementing FCM while increasing *c* and *m*. We performed separate FCM cluster analysis by gradually increasing c from 50 to 250 in increments of 50 (*c*= 50, 100, 150, 200 & 250) and specifying *m* = 1.05, 1.15, 1.25, 1.35, 1.45 & 1.55. For each FCM cluster analysis, we determined the overall mean of the membership values of a particular FCM cluster analysis (a single combination of *c* and *m*). We noted the number of genes included in clusters (not all genes cluster under all conditions) and the largest and smallest cluster size for each of the FCM cluster analyses.

Biological process GO term enrichment of each the clusters were tested using the GOEAST tool [68] assuming our experiment was a customized microarray platform. The p-value of GO ID enrichment was calculated as the hypergeometric probability of getting X genes (number of genes in each of the clusters) under the null hypothesis that they were selected randomly from the total pool of 3,281 genes. In order to control error rates for multiple hypothesis testing, the p-values were adjusted using Benjamini Hochberg method [69], where a false discovery rate (FDR)-adjusted *p*-value < 0.15 was considered significant.

### Upstream sequence analysis

#### Identification of upstream sequences

Whole genome sequence (v 4.2) and gene-predictions of the all protein-encoding genes for *Cryptosporidium parvum* were obtained from CryptoDB (http://cryptodb.org). Custom Perl scripts were used to extract upstream sequences. We defined the upstream region of a gene as 1 kb of sequence upstream of the ATG (few UTR sequences are known), or until an annotated gene is encountered on either strand, whichever sequence length is smaller. To exclude the possibility of including coding regions in this set due to misannotation, a BLASTX was performed against the NCBI nr database using the set of upstream sequences as the query. Upstream sequences that contained significant portions of 100% identity to coding sequences were pruned.

#### Identification of conserved motifs upstream of clustered genes

Upstream regions of genes present in each cluster were analyzed for *de novo* patterns using 3 pattern-finding algorithms: Multiple EM for Motif Elicitation (MEME) [70]; AlignACE [71] and Finding Informative Regulatory Elements (FIRE) algorithm [17].

MEME was run using the parameters minw=7, maxw=20, in two modes (zoops & anr) and the significant motifs (E-value >= 1e-01) for each cluster were examined. A background model is used by MEME to calculate the log likelihood ratio and statistical significance of the motif. The models used in this study were a zero-order Markov chain derived from all the non-coding sequences of *C. parvum*, as well as a zero-order Markov chain derived from all the coding sequences of *C. parvum*.

The AlignACE Gibbs-sampler motif finding algorithm parameters were set to seven aligned columns, 10 expected sites and GC%=27 (the background GC frequency of all the upstream sequence for *C. parvum)*. We used the motif comparison tool, STAMP [27] to compare the motifs identified by MEME and AlignACE. Those motifs that have a STAMP E-value less than 1e-05 were considered to be similar.

FIRE, a *de novo* motif discovery program, was implemented by specifying the motif seed length *k* as 5, 6, 7 and 8. Those motifs (statistically significant with a z-score > 4.0) on a robustness index ranging from 1 to 10 and also present in at least 60% of the upstream sequences of a cluster were considered significant in this study. FIRE was also run on all *C. parvum* coding sequences as a control.

#### Identification of conserved upstream motifs

Upstream regions for nine *Cryptosporidium* oocyst wall protein (COWP) genes; 105 genes belonging to clusters 7, 44 and 162 peaking primarily at 72 hours post-infection; and 68 *P. falciparum* and 60 *C. parvum* ribosomal protein genes were each separately mined for overrepresented motifs using MEME (max motif width 12 bp, 5 motifs max, mode = anr). Similarity of motifs to each other was determined via the STAMP tool [27].

### ApiAP2 domain binding site determination

N-terminal GST fusion proteins were made as previously described [13], using the pGEX4T-1 vector (GE Healthcare) and the predicted AP2 domains and flanking residues from cgd8_810 (the predicted domain spans from residues 584 to 637; residues 543–676 were tested) and the previously examined domain cgd2_3490 (the predicted domain spans from residues 341 to 394; residues 299–463 were tested) as a control [26]. Many flanking residues were included to ensure capture of the domain. The domain and flanking sequence were PCR-amplified and cloned into the BamHI restriction site in pGEX4T-1. Proteins were expressed and purified as in [26]. Briefly, *E. coli* BL21 (RIL Codon PLUS, Stratagene) cells were induced with 200 mM IPTG at 25°C. Proteins were then purified using Uniflow Glutathione Resin (Clontech) and eluted in 10 mM reduced glutathione, 50 mM Tris HCL, pH 8.0. Proteins were verified with western blots using an anti-GST antibody (Invitrogen), and purity was verified by silver stain. A minimum of two PBM experiments were performed with each purified protein construct to determine their binding specificities as previously described [13,14,26].

#### Additional files

The following additional data are available with the online version of this paper: an Excel spreadsheet with Supplementary Tables 1-4 (Additional file 1); a PDF detailing the 11 additional motifs and Supplementary Figures S1-S8 and their captions (Additional file 2).

## Competing interests

The authors declare that they have no competing interests.

## Authors’ contribution

JO, SJJ and JCK conceived and designed the experiments; JO & SJJ performed the experiments; JO, SJJ, and JCK analyzed the data and wrote the paper. All authors read and approved the final manuscript.

## Authors’ information

JO is currently located the University of South Florida and SJJ is currently located at Emory University. The work presented here was conceived and carried out while all authors were at the University of Georgia.

## Supplementary Material

Additional file 1 **Supplementary data tables. Tables S1-S4** which cover: 1) FCM cluster analysis parameter exploration; 2) GeneIDs associated with each cluster; Occurrences of all 25 overrepresented motifs; and 4) Co-occurrence of all 25 motifs upstream of 200 clusters. Click here for file

Additional file 2 **Supplementary figures. Figures S1-S7** describe additional motifs discovered in this study. They present the expression data, representative cluster profiles and cluster details associated with each motif. **Figure S8** presents the cluster profiles for all 200 clusters including membership values. Click here for file
