# Supplementary material for: Genome-wide upstream motif analysis of Cryptosporidium parvum genes clustered by expression profile
Source: BMC Genomics. 2013 Jul 29;14:516. doi: 10.1186/1471-2164-14-516 (PMC3734150; doi:10.1186/1471-2164-14-516)
Supplement: Additional file 2 — Supplementary figures. Figures S1-S7 describe additional motifs discovered in this study. They present the expression data, representative cluster profiles and cluster details associated with each motif. Figure S8 presents the cluster profiles for all 200 clusters including membership values. [file 1471-2164-14-516-S2.pdf]

Additional file 2: Supplementary materials

These supplementary materials accompany the manuscript "Genome-wide upstream motif and DNA-binding analysis of *Cryptosporidium parvum* genes clustered by expression profile" by Oberstaller et al. Supplementary Figures S1-S8 appear in this document. Supplementary Tables 1-4 appear in the spreadsheet Additional file 1.

Results

Motif 5, which is rich in G's and A's, is similar to the 5′-GAGA-3′ motif identified in *Drosophila* [1], as well as the NTBA (NucleoTide Biosynthesis-A) motif identified upstream of the nucleotide biosynthetic genes in *T. gondii* [2]. No proteins similar to the known GAGA-binding family of transcription factors are annotated in the *C. parvum* genome. The GAGA motif is found to be significantly overrepresented in the upstream regions of genes in 12 co-expressed gene clusters (totaling 127 genes) in the current study (Figure S1 A-D; Additional file 1, Table S3). Important GO terms enriched in these clusters are nucleosome assembly, DNA dependent transcription initiation, protein dephosphorylation and ubiquitin-dependent protein catabolic process.

Motifs 13, 16 and 20 are similar to the CCAAT-box (5′-CCAAT-3′) motif [3]. The *C. parvum* genome contains three putative CCAAT-binding transcription factors (Table 2). These motifs are overrepresented in the upstream regions of genes present in 21 gene clusters (228 genes) that are maximally expressed at any of the seven time points (Figure S2 A-D), with biological process enrichments in processes including ATP synthesis coupled proton transport, intracellular protein transport, oxidative phosphorylation, cytoskeletal organization and microtubule-based movement.

Motifs 10, 12, 15, 17, 18, and 19 comprise the family we term "Unknown set 1" and do not appear similar to any known *cis*-regulatory motifs. Several of these motifs are overrepresented upstream of single clusters (Figure S3 A-D). Motif 17 was found only in the promoter region of the genes present in cluster 6, which is enriched with ribosomal proteins. GO enrichment analysis of the genes in cluster 6 revealed highly significant enrichment of GO terms associated with gene expression, translation, translational elongation and tRNA aminoacylation. Motifs 18 and 19 are each conserved in the upstream region of clusters, 21 and 143 respectively. The genes in cluster 21 do not show any specific GO-enrichment. Genes in cluster 143 are enriched with ubiquitin-dependent protein catabolic process and protein dephosphorylation GO terms.

Motifs 9 and 24 comprise the "Unknown set 2" motif family, with the consensus sequence 5′-[C/T][C/T]T[A/G]CA-3′. Unknown set 2 motifs are found upstream of 15 clusters maximally expressed at any of the seven time points (Figure S4 A-D). Motifs 21, 22 and 25 are unrelated, do not have overt similarity to any known *cis*-regulatory motifs, and are overrepresented upstream of 3, 5, and 12 clusters respectively. Again, clusters containing these overrepresented motifs are expressed at any of the tested time points (Figure S5-S7 A-D). Major biological process GO terms found enriched in these clusters include nucleosome assembly (motif 21), translation (motif 22), regulation of gene expression, DNA-dependent transcription initiation, post-translational protein modification and protein dephosphorylation (motif 25). Finally, Figure S8 contains all 200 of the clustered expression profiles, 40 profiles per page.

Supplementary figures

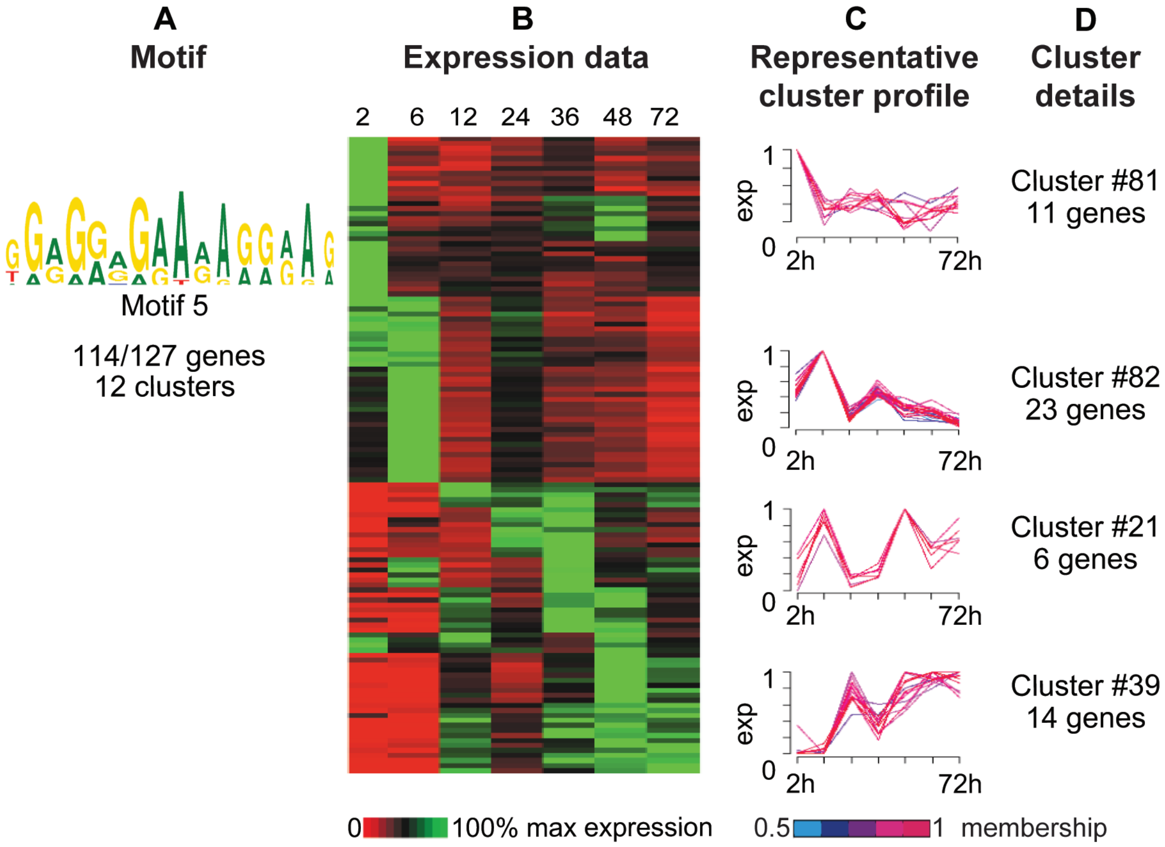

Figure S1. Data supporting identification of GAGA-like motifs. A. GAGA-like motifs. Motif name and total number of genes possessing each motif per total genes in all clusters where the motif is overrepresented are indicated. **B.** Expression data for 2, 6, 12, 24, 36, 48 and 72 hours post-infection for all genes from each cluster where the motif is overrepresented. Each row indicates a gene; rows are sorted first by cluster, then by peak expression at each time point. Gene IDs for genes associated with each cluster can be found in Additional file 1, Table S2. Expression is indicated on a scale of 0-100% of max for each gene. **C.** Four representative cluster profiles selected from the 12 clusters containing overrepresented GAGA-like motifs. Line colors for individual gene profiles indicate the membership values of that gene profile to the cluster ranging from 0.5 to 1. Each cluster profile is located next to the corresponding rows in the gene expression heatmap. **D.** Cluster number and total number of genes in each displayed representative cluster.

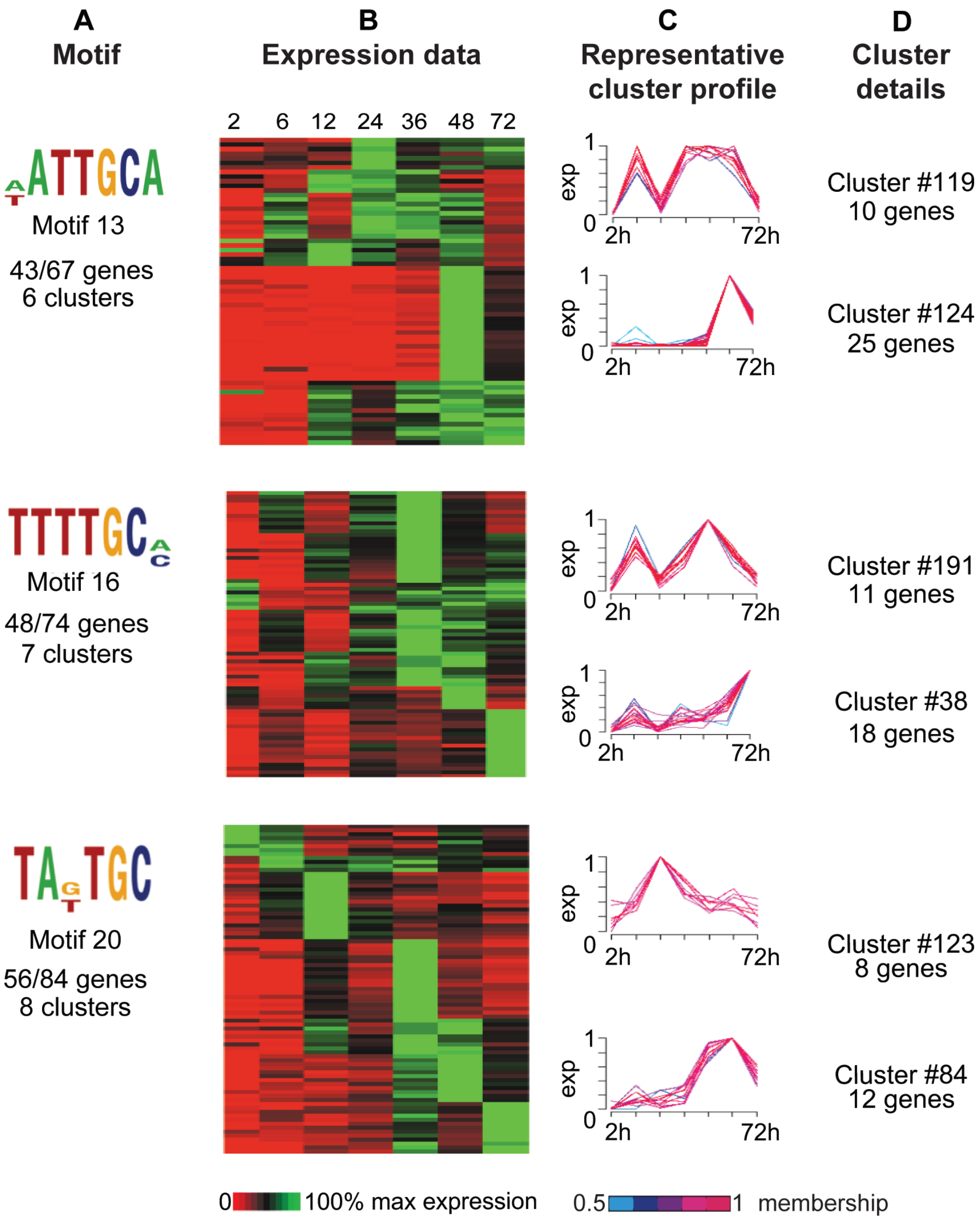

Figure S2. Data supporting identification of CAAT-box-like motifs. A. CAAT-box-like motifs (reverse complement). Motif name and total number of genes possessing each motif per total genes in all clusters where the motif is overrepresented are indicated. **B.** Expression data for 2, 6, 12, 24, 36, 48 and 72 hours post-infection for all genes from each cluster where the motif is overrepresented. Each row indicates a gene; rows are sorted first by cluster, then by peak expression at each time point. Gene IDs for genes associated with each cluster can be found in Additional file 1, Table S2. Expression is indicated on a scale of 0-100% of max for each gene. **C.** Six representative cluster profiles selected from the 21 clusters containing overrepresented CAAT-box-like motifs. Line colors for individual gene profiles indicate the membership values of that gene profile to the cluster ranging from 0.5 to 1. Each cluster profile is located next to the corresponding rows in the gene expression heatmap. **D.** Cluster number and total number of genes in each displayed representative cluster.

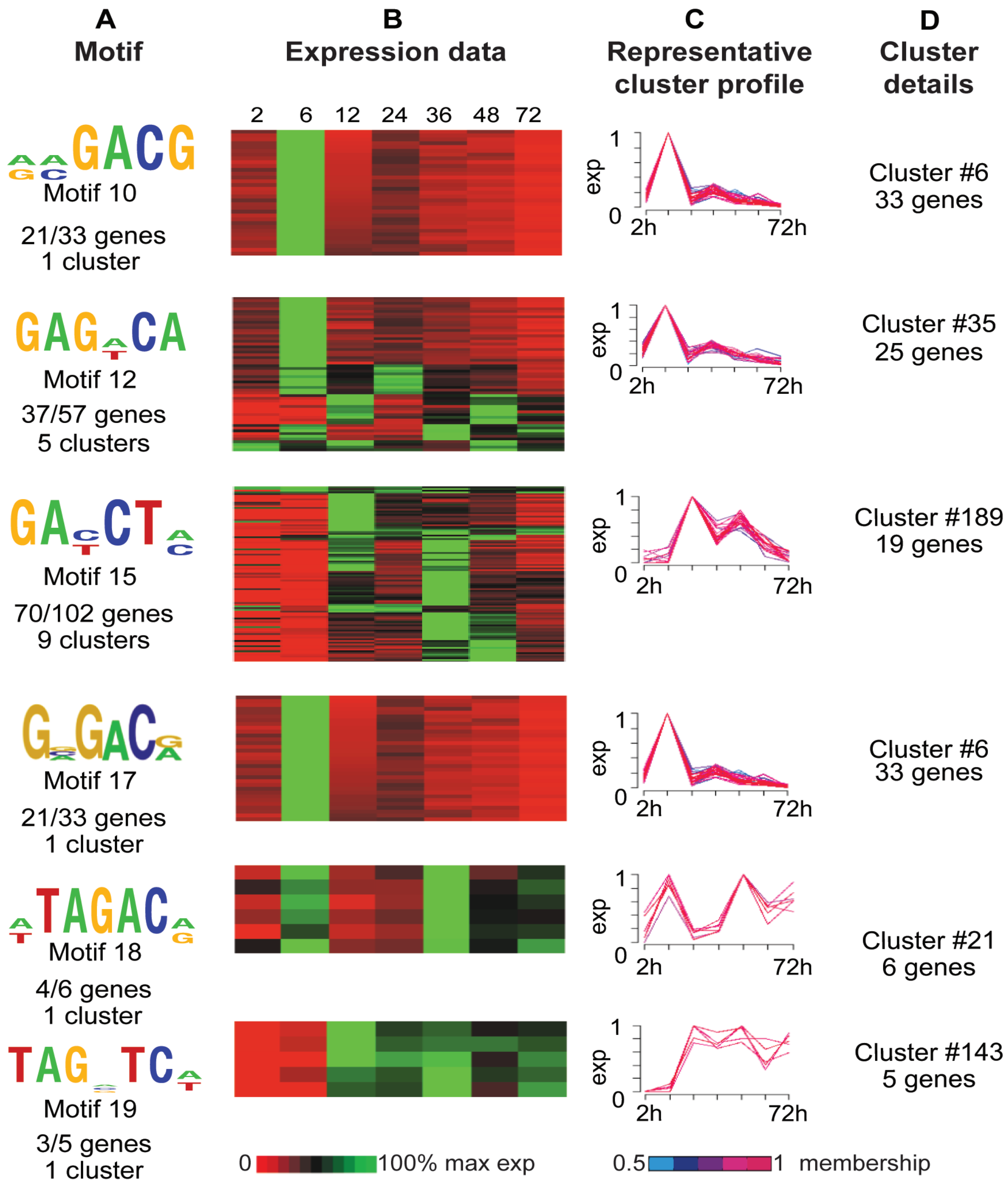

Figure S3. Data supporting identification of Unknown set 1 motifs. A. Unknown set 1 motifs. Motif name and total number of genes possessing each motif per total genes in all clusters where the motif is overrepresented are indicated. **B.** Expression data for 2, 6, 12, 24, 36, 48 and 72 hours post-infection for all genes from each cluster where the motif is overrepresented. Each row indicates a gene; rows are sorted first by cluster, then by peak expression at each time point. Gene IDs for genes associated with each cluster can be found in Additional file 1, Table S2. Expression is indicated on a scale of 0-100% of max for each gene. **C.** Six representative cluster profiles selected from the 18 clusters containing overrepresented Unknown set 1 motifs. Line colors for individual gene profiles indicate the membership values of that gene profile to the cluster ranging from 0.5 to 1. Each cluster profile is located next to the corresponding rows in the gene expression heatmap. **D.** Cluster number and total number of genes in each displayed representative cluster.

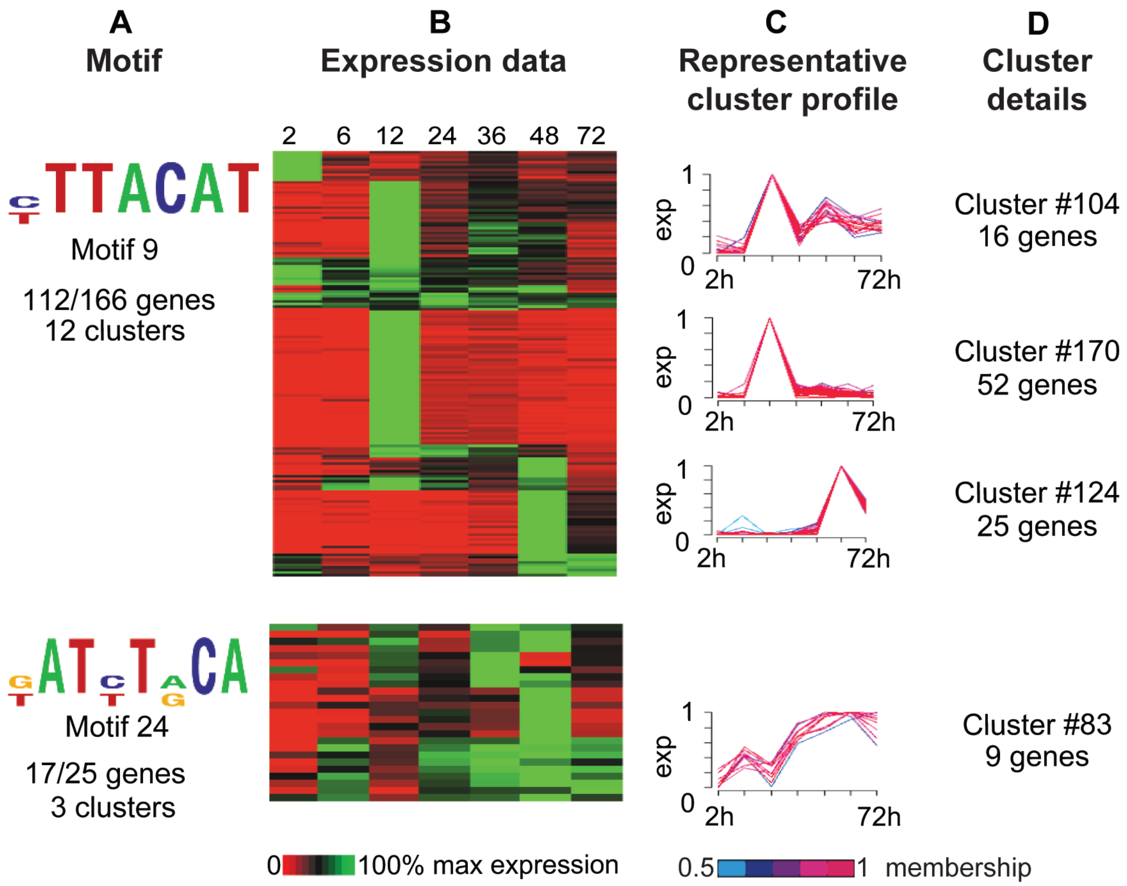

Figure S4. Data supporting identification of Unknown set 2 motifs. A. Unknown set 2 motifs. Motif name and total number of genes possessing each motif per total genes in all clusters where the motif is overrepresented are indicated. **B.** Expression data for 2, 6, 12, 24, 36, 48 and 72 hours post-infection for all genes from each cluster where the motif is overrepresented. Each row indicates a gene; rows are sorted first by cluster, then by peak expression at each time point. Gene IDs for genes associated with each cluster can be found in Additional file 1, Table S2. Expression is indicated on a scale of 0-100% of max for each gene. **C.** Four representative cluster profiles selected from the 15 clusters containing overrepresented Unknown set 2 motifs. Line colors for individual gene profiles indicate the membership values of that gene profile to the cluster ranging from 0.5 to 1. Each cluster profile is located next to the corresponding rows in the gene expression heatmap. **D.** Cluster number and total number of genes in each displayed representative cluster.

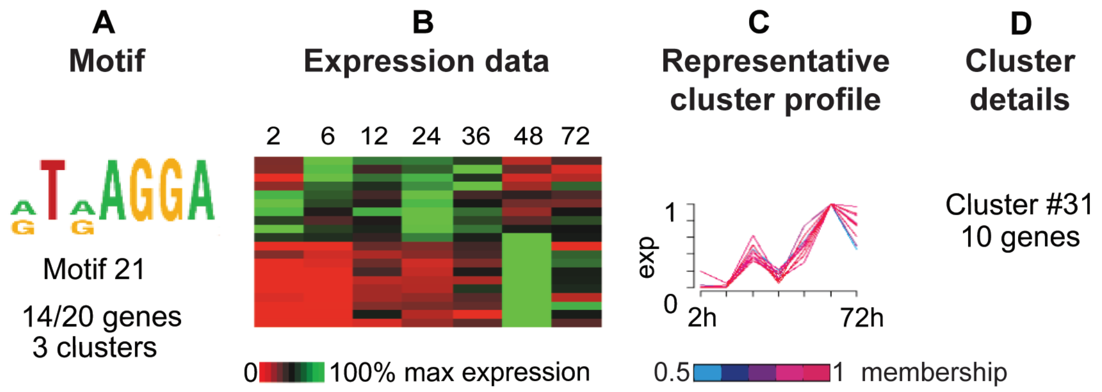

Figure S5. Data supporting identification of Unknown motif 21. A. Unknown motif 21. Total number of genes possessing the motif per total genes in all clusters where the motif is overrepresented is indicated. **B.** Expression data for 2, 6, 12, 24, 36, 48 and 72 hours post-infection for all genes from each cluster where the motif is overrepresented. Each row indicates a gene; rows are sorted first by cluster, then by peak expression at each time point. Gene IDs for genes associated with each cluster can be found in Additional file 1, Table S2. Expression is indicated on a scale of 0-100% of max for each gene. **C.** One representative cluster profile selected from the 3 clusters containing overrepresented Unknown motif 21 Line colors for individual gene profiles indicate the membership values of that gene profile to the cluster ranging from 0.5 to 1. Each cluster profile is located next to the corresponding rows in the gene expression heatmap. **D.** Cluster number and total number of genes in the displayed representative cluster.

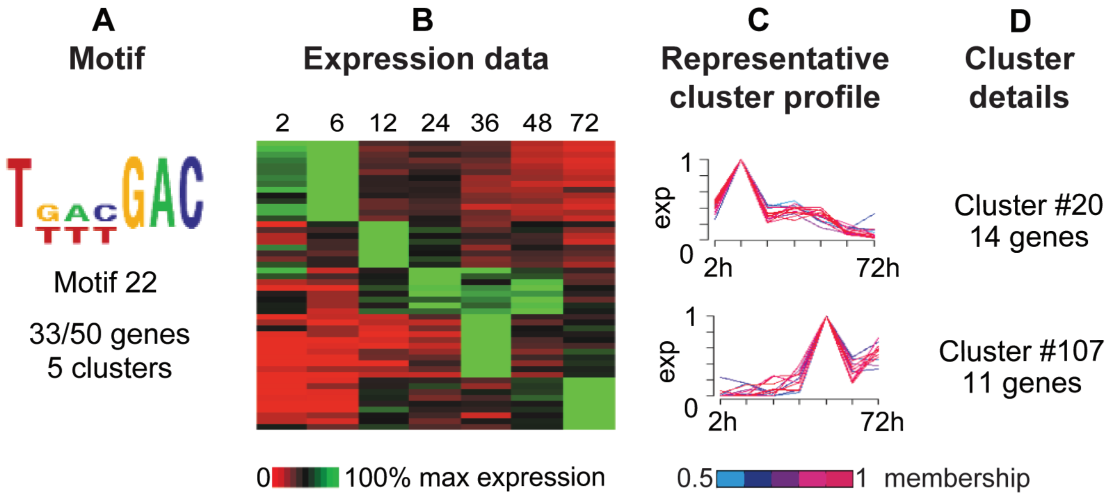

Figure S6. Data supporting identification of Unknown motif 22. A. Unknown motif 22. Total number of genes possessing the motif per total genes in all clusters where the motif is overrepresented is indicated. **B.** Expression data for 2, 6, 12, 24, 36, 48 and 72 hours post-infection for all genes from each cluster where the motif is overrepresented. Each row indicates a gene; rows are sorted first by cluster, then by peak expression at each time point. Gene IDs for genes associated with each cluster can be found in Additional file 1, Table S2. Expression is indicated on a scale of 0-100% of max for each gene. **C.** Two representative cluster profiles selected from the 5 clusters containing overrepresented Unknown motif 22. Line colors for individual gene profiles indicate the membership values of that gene profile to the cluster ranging from 0.5 to 1. Each cluster profile is located next to the corresponding rows in the gene expression heatmap. **D.** Cluster number and total number of genes in each displayed representative cluster.

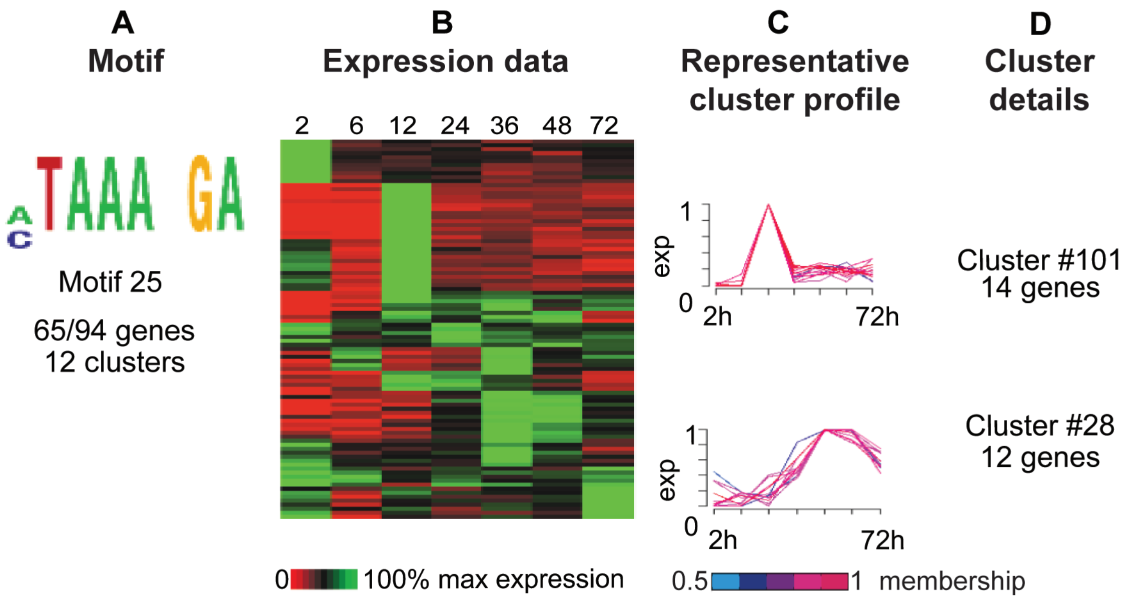

Figure S7. Data supporting identification of Unknown motif 25. A. Unknown motif 25. Total number of genes possessing the motif per total genes in all clusters where the motif is overrepresented is indicated. **B.** Expression data for 2, 6, 12, 24, 36, 48 and 72 hours post-infection for all genes from each cluster where the motif is overrepresented. Each row indicates a gene; rows are sorted first by cluster, then by peak expression at each time point. Gene IDs for genes associated with each cluster can be found in Additional file 1, Table S2. Expression is indicated on a scale of 0-100% of max for each gene. **C.** Two representative cluster profiles selected from the 12 clusters containing overrepresented Unknown motif 25. Line colors for individual gene profiles indicate the membership values of that gene profile to the cluster ranging from 0.5 to 1. Each cluster profile is located next to the corresponding rows in the gene expression heatmap. **D.** Cluster number and total number of genes in each displayed representative cluster.

Figure S8. Clustered expression profiles, clusters 1-200. Normalized expression levels for each gene on a scale of 0 (no expression) to 1 (max expression) at 2, 6, 12, 24, 36, 48 and 72 hours post-infection for each cluster. Line colors for individual genes in the profiles indicate the membership values of that gene profile to the overall cluster. The membership values range from 0.5 to 1. Red = 0.9-1; bright pink = 0.8-0.89; purple = 0.7-0.79; dark blue = 0.6-0.69; light blue = 0.5-.59.

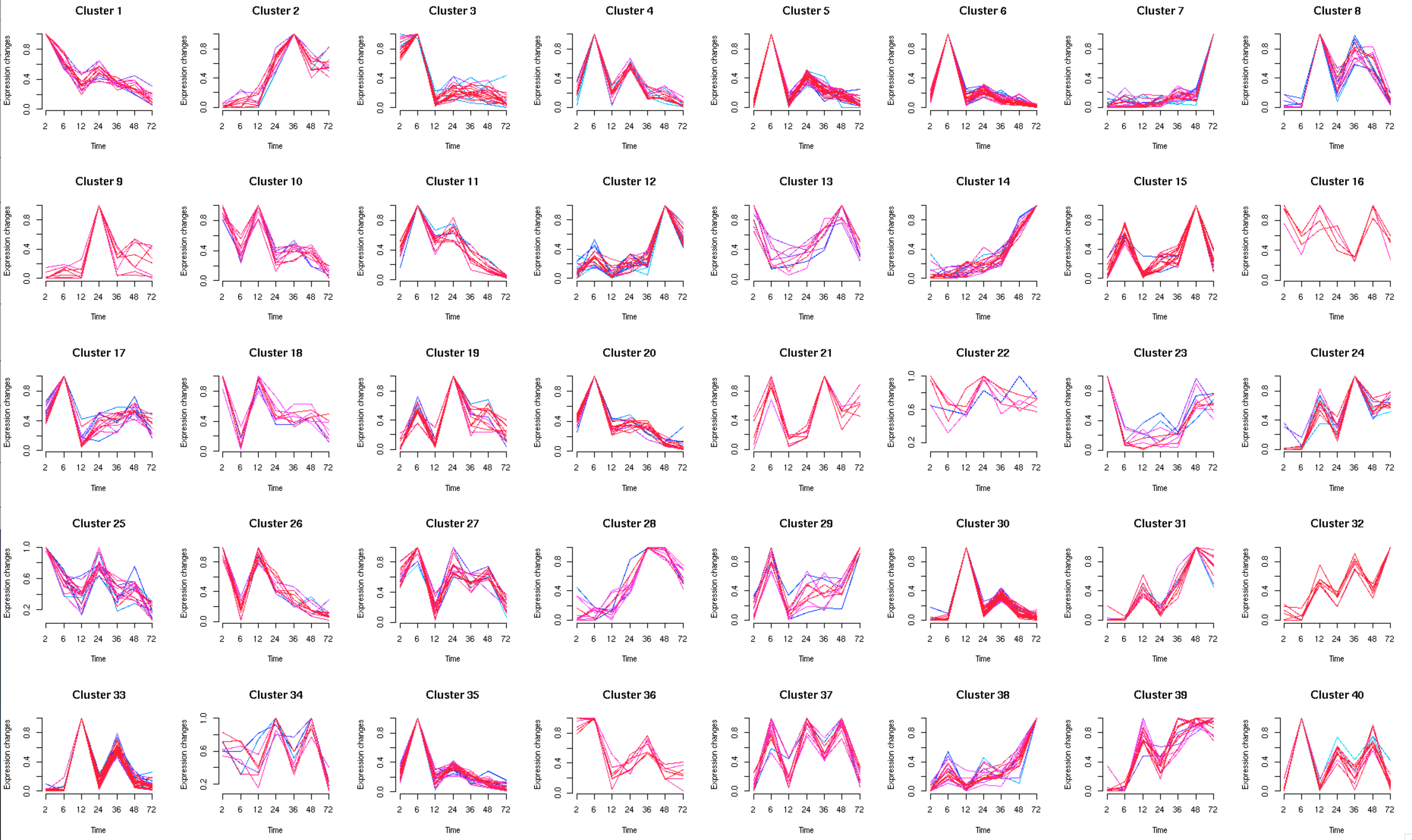
Cluster 1
Cluster 2
Cluster 3
Cluster 4
Cluster 5
Cluster 6
Cluster 7
Cluster 8
Cluster 9
Cluster 10
Cluster 11
Cluster 12
Cluster 13
Cluster 14
Cluster 15
Cluster 16
Cluster 17
Cluster 18
Cluster 19
Cluster 20
Cluster 21
Cluster 22
Cluster 23
Cluster 24
Cluster 25
Cluster 26
Cluster 27
Cluster 28
Cluster 29
Cluster 30
Cluster 31
Cluster 32
Cluster 33
Cluster 34
Cluster 35
Cluster 36
Cluster 37
Cluster 38
Cluster 39
Cluster 40
Expression changes
Time
2 6 12 24 36 48 72
0.0 0.4 0.8
0.2 0.6 1.0

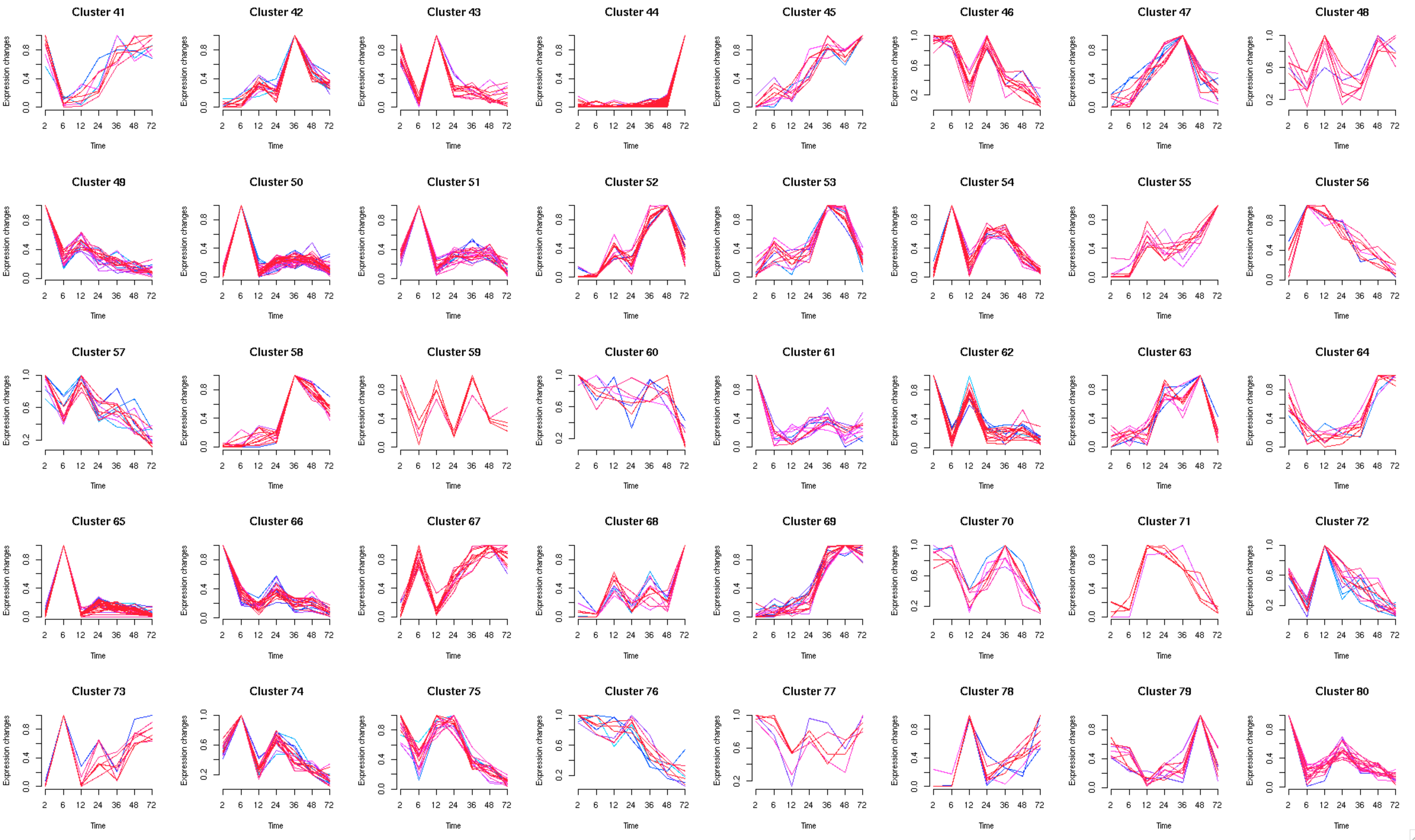
Cluster 41
Cluster 42
Cluster 43
Cluster 44
Cluster 45
Cluster 46
Cluster 47
Cluster 48
Cluster 49
Cluster 50
Cluster 51
Cluster 52
Cluster 53
Cluster 54
Cluster 55
Cluster 56
Cluster 57
Cluster 58
Cluster 59
Cluster 60
Cluster 61
Cluster 62
Cluster 63
Cluster 64
Cluster 65
Cluster 66
Cluster 67
Cluster 68
Cluster 69
Cluster 70
Cluster 71
Cluster 72
Cluster 73
Cluster 74
Cluster 75
Cluster 76
Cluster 77
Cluster 78
Cluster 79
Cluster 80
Expression changes
Time
2 6 12 24 36 48 72

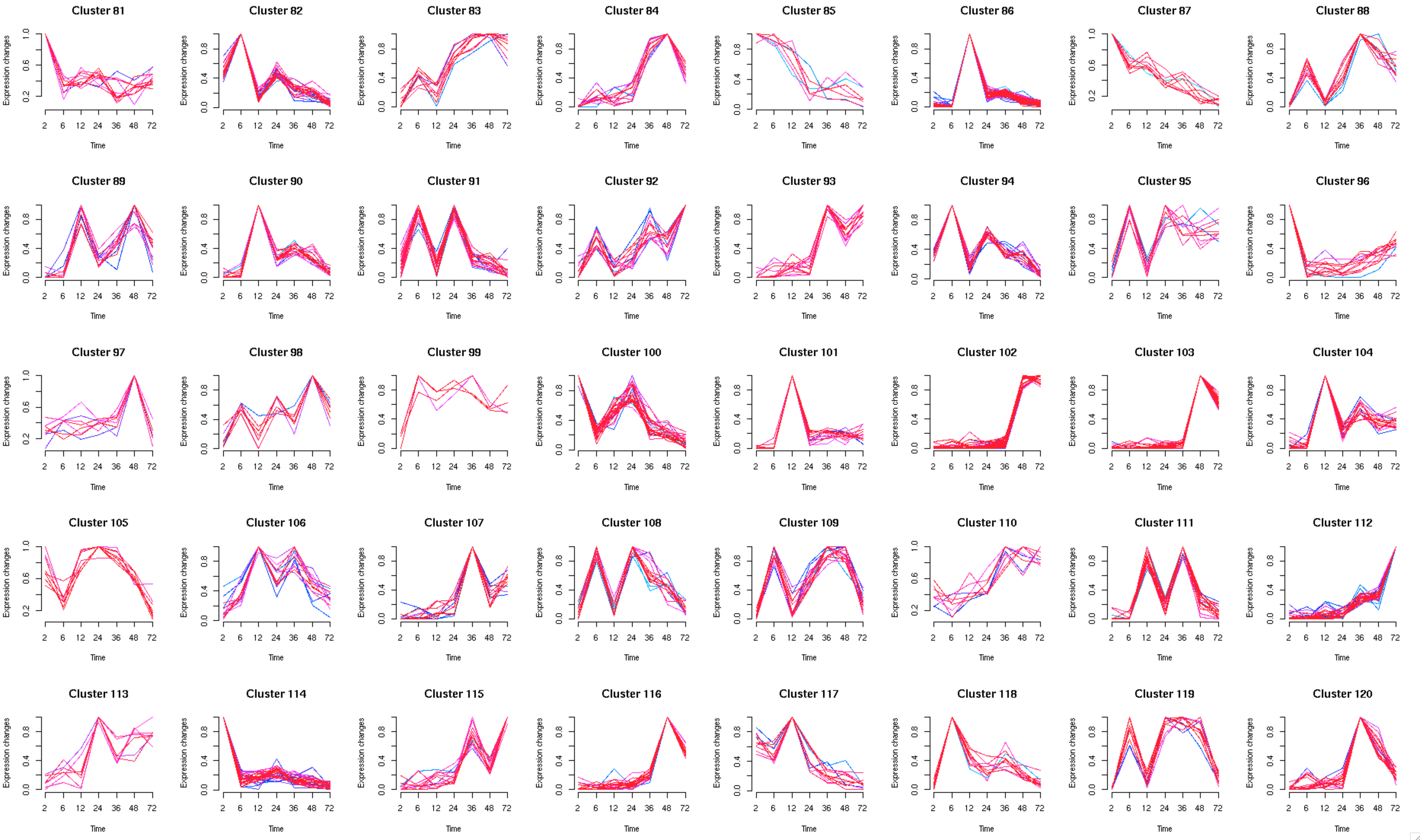
Cluster 81
Cluster 82
Cluster 83
Cluster 84
Cluster 85
Cluster 86
Cluster 87
Cluster 88
Cluster 89
Cluster 90
Cluster 91
Cluster 92
Cluster 93
Cluster 94
Cluster 95
Cluster 96
Cluster 97
Cluster 98
Cluster 99
Cluster 100
Cluster 101
Cluster 102
Cluster 103
Cluster 104
Cluster 105
Cluster 106
Cluster 107
Cluster 108
Cluster 109
Cluster 110
Cluster 111
Cluster 112
Cluster 113
Cluster 114
Cluster 115
Cluster 116
Cluster 117
Cluster 118
Cluster 119
Cluster 120
Expression changes
Time
2 6 12 24 36 48 72

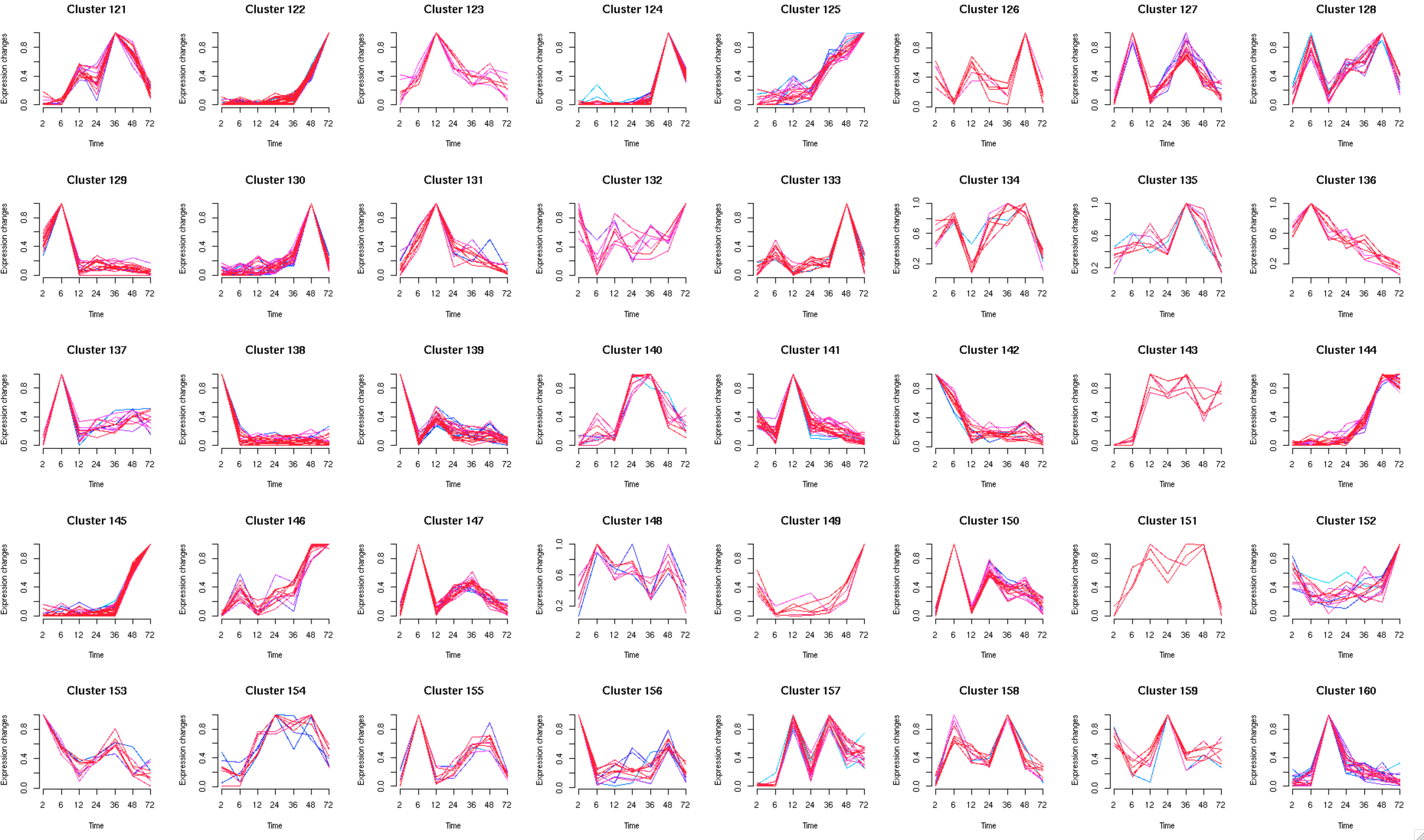
Cluster 121
Cluster 122
Cluster 123
Cluster 124
Cluster 125
Cluster 126
Cluster 127
Cluster 128
Cluster 129
Cluster 130
Cluster 131
Cluster 132
Cluster 133
Cluster 134
Cluster 135
Cluster 136
Cluster 137
Cluster 138
Cluster 139
Cluster 140
Cluster 141
Cluster 142
Cluster 143
Cluster 144
Cluster 145
Cluster 146
Cluster 147
Cluster 148
Cluster 149
Cluster 150
Cluster 151
Cluster 152
Cluster 153
Cluster 154
Cluster 155
Cluster 156
Cluster 157
Cluster 158
Cluster 159
Cluster 160
Expression changes
Time
2 6 12 24 36 48 72
0.0 0.4 0.8

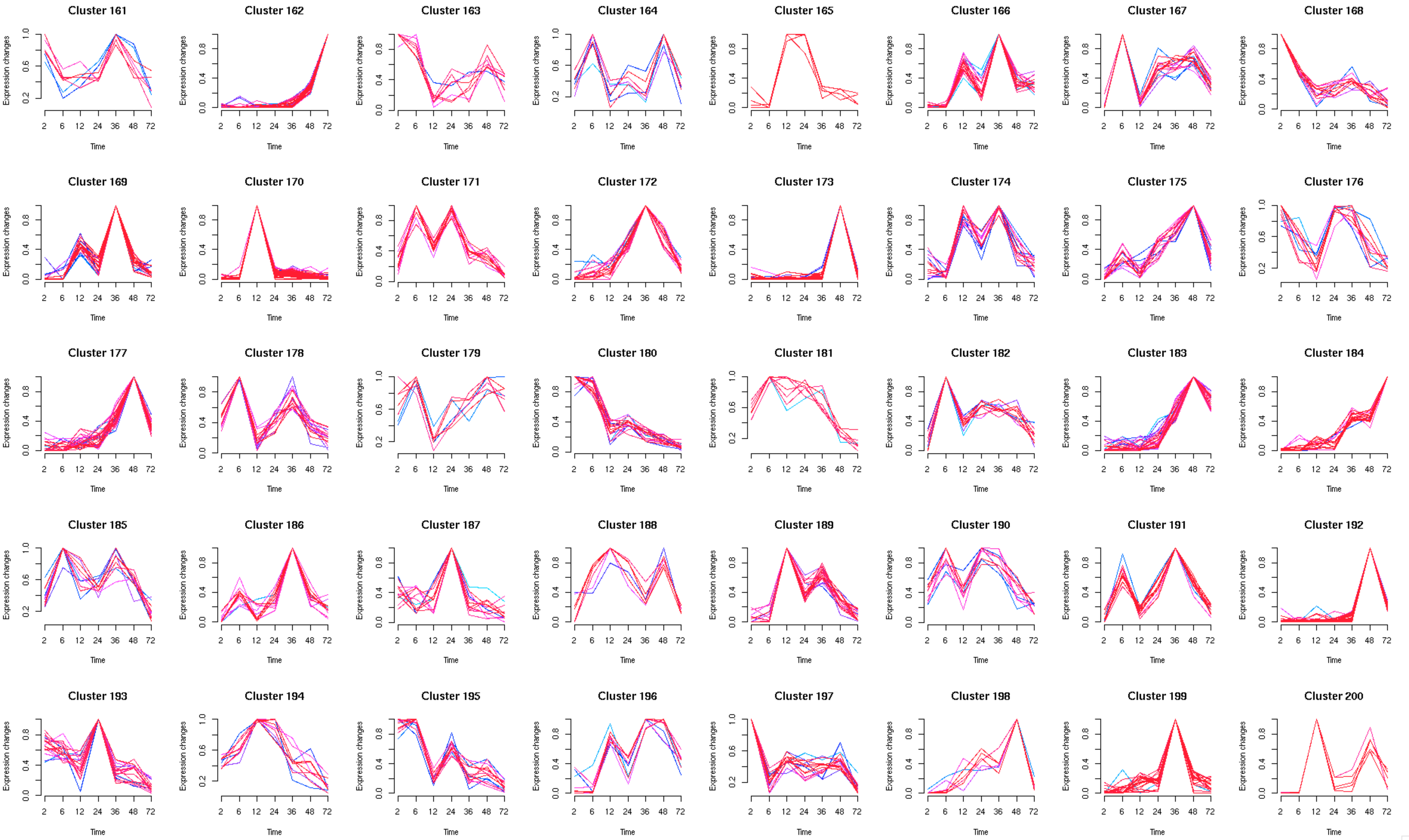
Cluster 161
Cluster 162
Cluster 163
Cluster 164
Cluster 165
Cluster 166
Cluster 167
Cluster 168
Cluster 169
Cluster 170
Cluster 171
Cluster 172
Cluster 173
Cluster 174
Cluster 175
Cluster 176
Cluster 177
Cluster 178
Cluster 179
Cluster 180
Cluster 181
Cluster 182
Cluster 183
Cluster 184
Cluster 185
Cluster 186
Cluster 187
Cluster 188
Cluster 189
Cluster 190
Cluster 191
Cluster 192
Cluster 193
Cluster 194
Cluster 195
Cluster 196
Cluster 197
Cluster 198
Cluster 199
Cluster 200
Expression changes
Time
2 6 12 24 36 48 72

References

1. Biggin MD, Tjian R: **Transcription factors that activate the Ultrabithorax promoter in developmentally staged extracts.** *Cell* 1988, **53:**699-711.
2. Mullapudi N, Joseph SJ, Kissinger JC: **Identification and functional characterization of *cis*-regulatory elements in the apicomplexan parasite *Toxoplasma gondii*.** *Genome Biol* 2009, **10:**R34.
3. Maity SN, de Crombrugghe B: **Role of the CCAAT-binding protein CBF/NF-Y in transcription.** *Trends in Biochemical Sciences* 1998, **23:**174-178.
